# Context-dependent feature modulation shapes human decision policies in approach–avoidance conflicts

**DOI:** 10.1371/journal.pcbi.1014793

**Published:** 2026-09-30

**Authors:** Sergej A. E. Golowin, Niall W. Duncan, Faizan Shaikh, Christoph W. Korn

**Affiliations:** 1 Section Social Neuroscience, Department of General Psychiatry, University of Heidelberg, Heidelberg, Germany; 2 Graduate Institute of Mind, Brain and Consciousness, Taipei Medical University, Taipei, Taiwan; 3 Department of Mathematics and Computer Science, Faculty of Health, Science and Technology, Karlstad University, Karlstad, Sweden; Chinese Academy of Sciences, CHINA

## Abstract

Sequential decisions often involve trade-offs between immediate rewards and future risks, particularly in approach–avoidance contexts. While such behavior is commonly framed in terms of optimization principles, it remains unclear how decision processes adapt across contexts. We developed a sequential foraging task in which participants made binary choices under probabilistic reward and predation risk (threat). The design dissociated reward probability from threat and enabled formal comparison of policies based on decision-relevant features (e.g., reward and threat probabilities), a multi-feature policy, and a mathematically optimal policy derived from a fully observable Markov Decision Process. Crucially, the task included two implicitly signaled conditions – approach and avoidance – that differed in how reward and threat information jointly shaped the optimal policy. In both conditions, higher reward probabilities were associated with higher predation risk. However, the balance between these competing factors differed across environments, creating contexts in which the optimal policy either favored approaching or avoiding the higher-threat option. Behavior was analyzed using hierarchical Bayesian models capturing both individual decision features and optimal action values, as well as their modulation by task context.

Avoidance contexts selectively altered the weighting of decision features, with reduced reliance on reward probability. In addition, choices showed evidence for increased alignment with optimal state–action values, even after accounting for single feature policies within a shared model. This indicates that behavior more strongly reflected the value structure of the environment under avoidance, consistent with enhanced integration of decision-relevant features.

These findings suggest that adaptive behavior in sequential decision-making arises from context-dependent feature reweighting alongside increased alignment with integrated value signals. This provides a parsimonious account of how humans adjust decision-making under threat, without requiring assumptions about discrete strategy shifts.

## Author summary

Many everyday decisions require balancing potential rewards against possible risks. People must also consider how current choices affect future outcomes, for example when pursuing a job opportunity, making an investment, or entering an uncertain social situation. However, it remains unclear how decision-making adapts when circumstances change. To address this question, we developed a computer-based foraging game in which participants repeatedly chose between a potentially rewarding but risky option and a safer alternative. The task created environments in which the balance between reward and threat favored either approaching or avoiding risk. Because the task was based on a mathematical model of the game, we could compare participants’ choices with the choices that would maximize long-term success. We found that behavior was best explained by the combined influence of several simple factors, including the likelihood of reward, the likelihood of threat, and participants’ resources. Importantly, avoidance contexts reduced reliance on reward-related information while increasing alignment with the strategy that maximized long-term success. Rather than switching between entirely different decision strategies, participants adapted by changing how much weight they gave to different types of information. These findings suggest that flexible decision-making arises from adjusting the importance of available information as environmental demands change.

## 1 Introduction

When deciding whether to pursue a job opportunity, approach a social interaction, or invest in a financial asset, humans often face sequences of interdependent choices in which current actions shape future opportunities. Many such decisions additionally involve approach–avoidance conflicts (AAC), requiring individuals to balance potential rewards against possible threats or aversive outcomes [1,2]. Sequential choice naturally gives rise to accept–reject decisions [3], in which individuals must evaluate whether potential rewards outweigh associated costs or risks, closely paralleling approach–avoidance conflict. However, approach–avoidance conflict has primarily been examined in static paradigms, whereas sequential models have focused on reward-based decisions without explicit incorporation of threat [4–6]. To address this gap, we developed a sequential choice paradigm in which reward and threat relationships differed across approach and avoidance contexts.

Integrating sequential decision-making with AAC is particularly valuable because adaptive behavior requires both anticipating future consequences and balancing competing gains and risks. Sequential choice paradigms provide a powerful framework for studying these processes because they allow behavior to be analyzed in terms of decision policies, that is, rules mapping states onto actions over time. Such policies may depend either on integrated value computations (e.g., evaluating whether accepting a risky opportunity is worthwhile in the long run) or on simpler decision-relevant features derived from observable environmental cues, such as reward probability or threat risk. In this sense, individual features can guide behavior through simple feature-based decision rules (e.g., higher reward probability → greater likelihood of accepting an offer). Policies based on integrated values, on the other hand, can be formalized within Markov Decision Processes (MDPs), which specify value-maximizing behavior across temporally extended choice sequences [7]. Within such frameworks, optimal policies can be derived using dynamic programming (Fig 1), yielding state–action values (*Q*-values) that quantify the expected long-term value of alternative actions and provide a normative benchmark against which observed behavior can be evaluated. Contemporary accounts of adaptive decision-making frequently emphasize integrated value representations that combine multiple sources of decision-relevant information [8–14]. Importantly, behavior need not rely on explicit value maximization to appear near-optimal. Similar adaptive behavior may also emerge through selective weighting of informative decision features, that is, through changes in how strongly decision-relevant features influence choice, allowing adaptive policies to be approximated without explicit computation of exhaustive value representations. Such lower-dimensional policy representations may provide a computationally scalable and flexible basis for adaptation [15]. Distinguishing between behavior that arises from integrated value representations and behavior that emerges through adaptive weighting of decision-relevant features is important because superficially similar adaptive behavior may arise from fundamentally different computational mechanisms, with distinct implications for theories of decision-making in psychology and economics.

**Fig 1:**
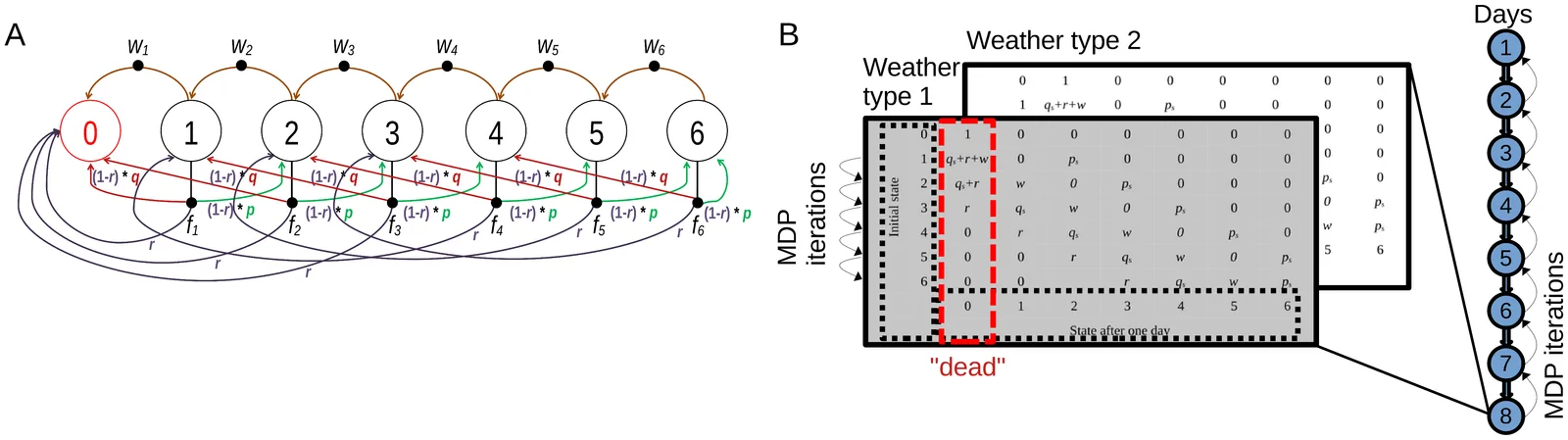
Illustration of Markov Decision Process (MDP) computation using backward induction (dynamic programming). **(A)** Transition graph between energy states for two actions: “w” (waiting) and “f” (foraging). The goal was to stay alive, so transitions to the absorbing zero-energy state (in red) incur a reward of –1, while all other transitions have a reward of 0. Crucially, transitions between states are governed by the probability of gain (p with q=1−p) and the risk of threat **(r)**, which determine how likely actions are to lead to different future energy states. **(B)** Backward induction illustrated schematically. Value updates proceed from the last to the first time-point (“days”), iteratively combining expected outcomes and selecting the higher-valued action at each step. State–action values (Q-values) represent the maximized expected reward of each action in a given state, integrating future consequences.

Within AAC, contextual variation emerges from how reward and threat jointly determine the long-term value of available actions. In some situations, it may be more favorable to take (or approach) higher risks imposed by a potential threat, whereas in other situations it is better to avoid threats. Such differences may place distinct demands on adaptive behavior by altering which decision features are most informative for guiding choice. For example, in an environment favoring threat approach, reward-related information may be more important, whereas environments involving stronger reward–threat trade-offs may increase reliance on threat-related features. Recent accounts suggest that this flexibility may arise not through switches between entirely distinct decision strategies, but through context-dependent changes in reliance on specific decision features [4–6,16]. Under this view, approach and avoidance contexts may shape behavior by selectively modulating the relative influence of specific decision features, such as reward probability or threat risk, while preserving a shared underlying computational process.

Despite their conceptual overlap, approach–avoidance conflict and sequential decision-making have largely been studied in isolation. AAC has primarily been examined in static paradigms, whereas sequential models have focused on reward-based decisions without explicit incorporation of threat [4–6]. Foraging paradigms provide a natural framework for integrating these perspectives because they capture how organisms adapt behavior across sequentially unfolding trade-offs through repeated decisions to exploit current opportunities or defer action in favor of potentially better alternatives [17–20]. By manipulating the relationship between reward and threat, such paradigms can generate distinct approach and avoidance contexts within a sequential decision framework, allowing adaptive behavior to be examined under systematically varying environmental demands. This framework further allows investigation of whether contextual adaptation primarily emerges through changes in integrated value representations or through selective modulation of individual decision features. Accordingly, the current work raises two key questions: (1) whether approach and avoidance contexts differ in the extent to which behavior aligns with optimal policies, and (2) whether such differences can be explained by context-dependent shifts in reliance on specific decision features.

To address these questions, we developed a sequential foraging task framed as a game and adapted from earlier virtual foraging paradigms [21,22], in which participants made binary choices under probabilistic outcomes reflecting competing risks of starvation and predation (Fig 2). The task was designed according to a fully specified MDP, allowing us to mathematically derive the optimal policy as a normative benchmark. Participants repeatedly chose between foraging and waiting in order to survive within the game, with choice outcomes determined by reward probability (food gain) and threat risk (i.e., the probability of a life-threatening predator encounter). Crucially, both reward and threat were defined probabilistically, allowing us to manipulate their relationship while keeping the task structure constant. In both conditions, weather types associated with greater reward opportunities also tended to involve greater threat. However, the balance between these competing factors differed across environments, creating contexts in which the optimal policy either favored approaching or avoiding the higher-threat option. This design allowed us to examine how environmental context alters the influence of reward- and threat-related decision features on adaptive behavior.

**Fig 2:**
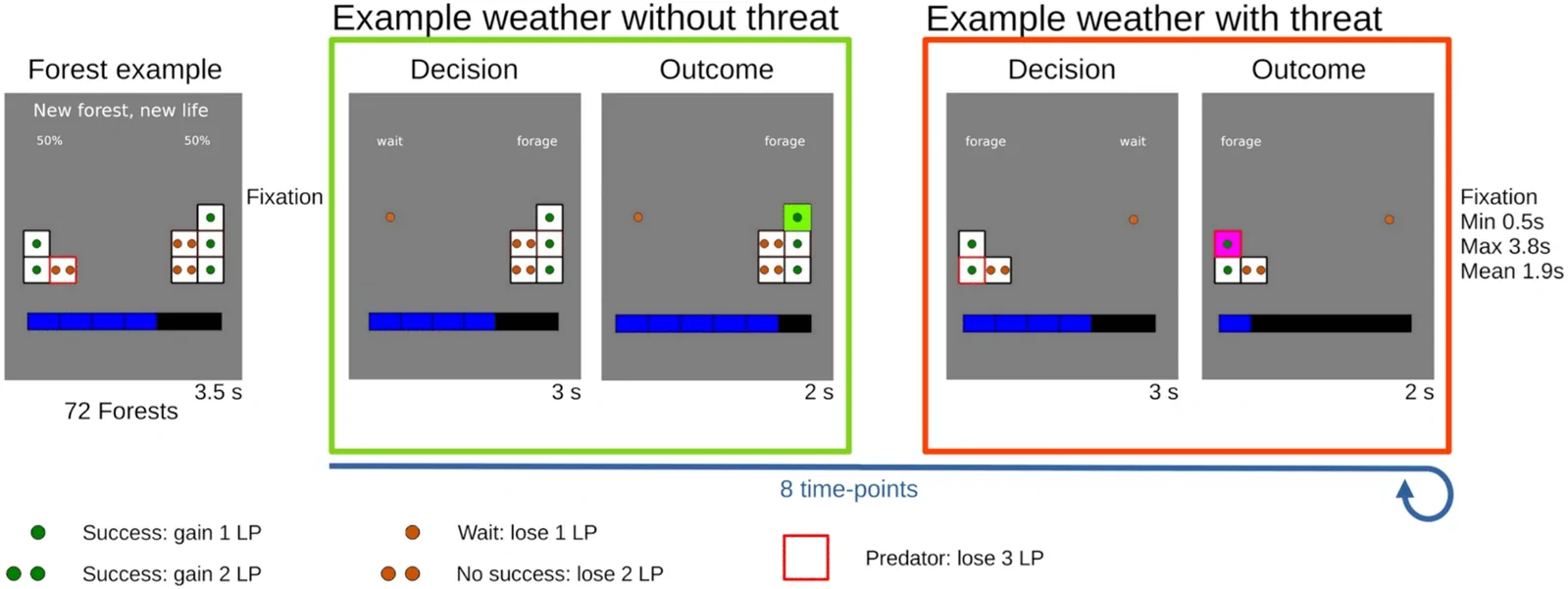
The Hunter-Gatherer Game with Predator. See Methods (Section 4.3, “Description of the Hunter–Gatherer Task With Predators”) for complete task details. Participants completed 72 sequential foraging environments (“forests”), each consisting of 8 consecutive time-points (trials). The goal of the game was to “survive” by keeping discrete energy points (maximum = 6) from depleting. At the beginning of a forest, energy levels were randomly set to 4 or 5. Each forest had two weather types displayed before the decision trials started. Each weather specified the probabilities of obtaining food, failing to forage, and (in threat environments) encountering a predator. At each time point, one weather type was randomly selected and participants chose between **foraging** and **waiting**. Successful foraging increased energy by 1–2 points, unsuccessful foraging decreased energy by 2 points, and waiting incurred a guaranteed loss of 1 energy point. In threat environments, predator encounters caused an additional loss of 3 energy points. The blue bar indicates the participant’s current energy level. The figure illustrates a forest overview (left), an example trial without predator threat (center), and an example trial with predator threat (right).

In summary, the current work examines whether contextual adaptation in sequential approach–avoidance conflict is reflected in changes in the degree to which choices align with the optimal policy and whether such adaptation can be explained by selective modulation of individual decision features. By jointly modeling behavior using optimal-policy and feature-based approaches, we test whether approach and avoidance contexts differentially influence the relationship between choices, integrated value signals, and task-relevant decision features.

## 2 Results

### 2.1 Task structure and behavioral regime

We tested 29 participants (15 female; mean age = 23.93 years, SD = 3.73), each completing up to 576 trials (72 forests × 8 days). On average, participants completed 361.45 trials (SD = 23.22; range = 292–411), yielding 10,270 decisions in total. Missed responses were rare (M = 0.02, SD = 0.03 trials), indicating high task engagement.

The task comprised two conditions: approach forests and avoidance forests, which differed in how reward and threat information jointly shaped the optimal decision policy. In both conditions, weather types associated with greater reward opportunities also tended to involve greater threat. However, the balance between these competing factors differed across environments, creating contexts in which the optimal policy either favored approaching or avoiding the higher-threat option. Descriptively, mean food-finding probability was lower in approach forests (0.40±0.17) than in avoidance forests (0.47±0.13), while predation risk was comparable across conditions (approach: 0.21±0.11; avoidance: 0.19±0.22). Consequently, joint success probability was higher in avoidance forests (0.37±0.12) than in approach forests (0.30±0.11). Despite these differences in environmental structure, survival rates did not differ significantly between conditions (t=−0.89, *p* = .38), providing no evidence that one condition produced substantially higher failure rates than the other. Bootstrapping using 10,000 resamples indicated only a small difference in predation-risk distributions between conditions (95% CI [0.007, 0.02]), suggesting that the practical magnitude of this difference was negligible.

Designing sequential choice tasks is challenging because action values depend on transition dynamics that cannot be directly controlled. We therefore constructed environments that avoided strongly favoring either action, minimizing both trivial and indifferent choices. To characterize the resulting decision space independently of participants’ behavior, we analyzed the distribution of theoretical state–action value differences ($\Delta Q=Q_{forage}-Q_{wait}$) across all states of all task environments. These ΔQ-values were derived from the optimal policy of the fully specified MDP underlying the task and served as a measure of choice difficulty. Positive ΔQ-values favor foraging, negative values favor waiting, and values near zero indicate little difference in long-term value between actions and therefore correspond to more difficult decisions. As shown in Fig 3A, most decisions clustered around ΔQ=0 (−0.2<ΔQ<0.2), indicating that the task predominantly sampled meaningful trade-offs, whereas truly indifferent choices were rare (3% of trials). This pattern was highly similar across approach and avoidance conditions (Fig 3B and 3C), suggesting comparable theoretical difficulty across contexts. Consistent with this, a Kolmogorov–Smirnov test revealed only a small difference between the ΔQ distributions (D = 0.04), despite statistical significance driven by the large number of theoretically possible states (*p* < .01). Together, these results indicate that the task predominantly sampled difficult but non-indifferent decisions while avoiding large numbers of trivial choices. Importantly, choices that more closely aligned with the action favored by ΔQ were associated with greater behavioral success (Fig 3D).

**Fig 3:**
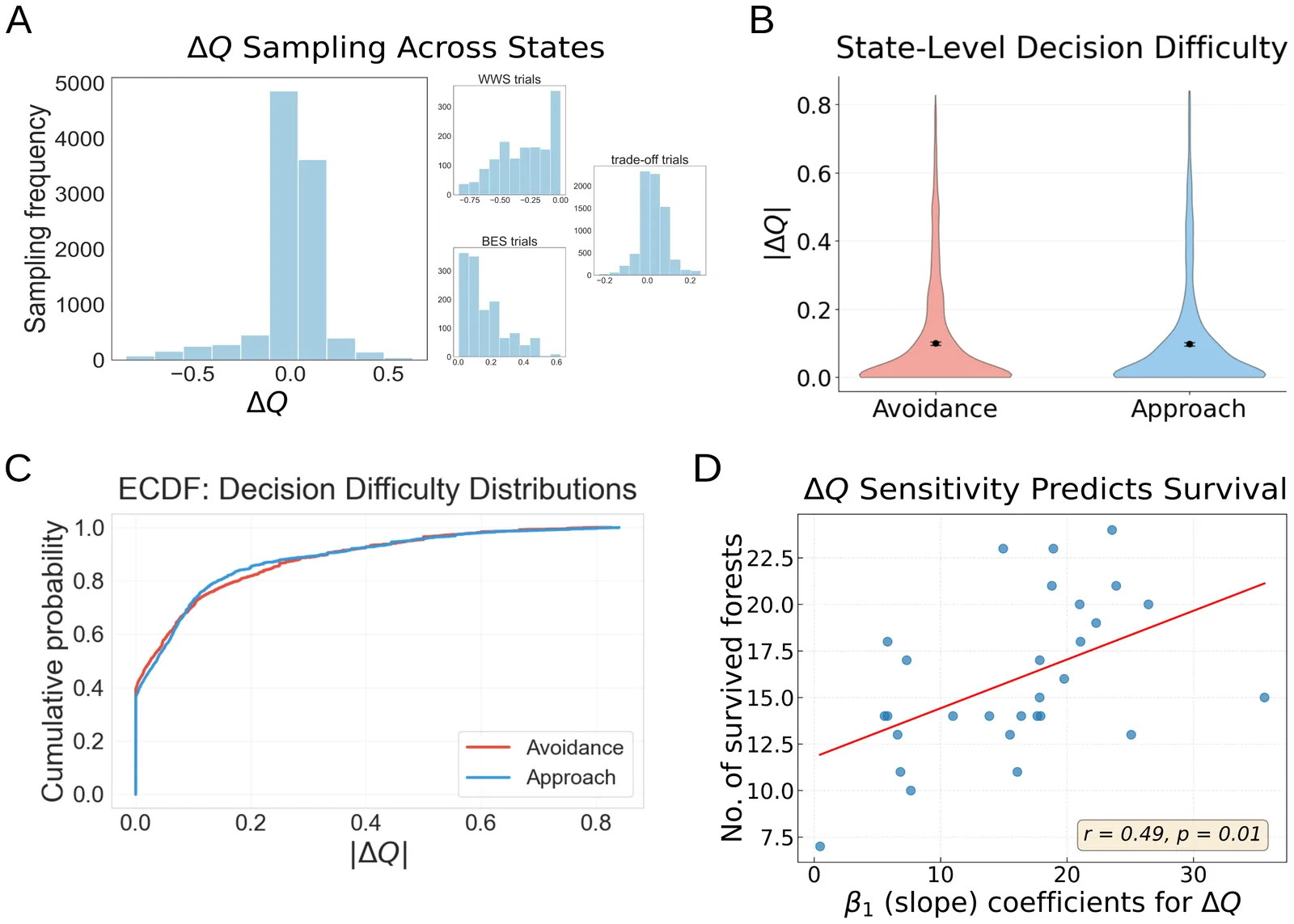
Structure of the decision space and behavioral relevance of optimal policy. **(A)** Distribution of the optimal policy value differences ($\Delta Q=Q_{forage}-Q_{wait}$) derived from the Markov Decision Process (MDP), quantifying choice difficulty. Values near zero indicate weak preference between actions, whereas larger absolute values reflect clearer optimal choices. The distribution is concentrated around zero, indicating that most trials involve finely balanced trade-offs. Insets illustrate example distributions for different trial types. **(B)** State-level decision difficulty (|ΔQ|) across conditions for the entire theoretical MDP grids (all possible state–action values). Violin plots show similar distributions for avoidance and approach conditions, indicating comparable overall task difficulty. Points denote mean values. **(C)** Empirical cumulative distribution functions (ECDFs) of |ΔQ| for approach and avoidance conditions. The strong overlap between curves indicates highly similar difficulty distributions across contexts. Although the Kolmogorov–Smirnov test was statistically significant (D = 0.04, p < 0.01), the small effect size suggests that differences between conditions are negligible. **(D)** Relationship between alignment with optimal policy value differences (ΔQ) and behavioral success. Each point represents a participant; the x-axis shows the individual slope ($\beta_{1}$) relating ΔQ to choice, and the y-axis shows the number of successfully completed forests. Greater alignment with optimal values is associated with higher performance (r = 0.49, p = 0.01), supporting the behavioral relevance of the MDP-derived benchmark.

To further evaluate whether the two conditions differed in overall processing demands, we compared log-transformed response times (RTs) between approach and avoidance contexts. There was no difference in RTs between conditions (Mann–Whitney *U* = 12908, *p* = .459). We next examined whether response times varied as a function of decision difficulty by regressing RTs on absolute state–action value differences (|ΔQ|), condition, and their interaction. No effects of |ΔQ| (*p* = .410), condition (*p* = .726), or their interaction (*p* = .464) were observed. Together with the comparable ΔQ distributions across conditions, these findings suggest that the approach and avoidance environments imposed similar overall processing demands and did not differ systematically in difficulty.

### 2.2 Model inference

Our task allowed us to derive the optimal policy using an MDP and to specify feature-based models of varying complexity (summarized in Table 1). Simple feature-based models relied on isolated decision variables such as weather or predator risk, whereas more complex models integrated multiple features into unified value representations, such as expected gain or the optimal policy, which require more computationally demanding evaluations. To bridge these extremes, we implemented a multi-feature policy that combines a small set of decision features capturing both environmental transition structure and state-dependent action constraints. Environmental features such as success probability (p(gain)) and threat risk (r(threat)) directly determine the transition dynamics of the MDP and therefore specify how current actions influence future states. In contrast, state-dependent features such as binary energy state (BES) and wait when safe (WWS) capture boundary conditions under which actions become either necessary or safely avoidable. We refer to these as boundary conditions because they reflect extreme environmental states associated with near-deterministic policies (e.g., foraging when starving). By combining these components, the multi-feature policy provides a structured, low-dimensional approximation of the optimal policy that captures both environmental dynamics and internal resource constraints without requiring explicit forward planning. This formulation allows us to test whether adaptive behavior is better explained by structured feature integration, isolated decision variables, or exhaustive MDP-based evaluations. Importantly, the resulting model space allows us to determine whether apparent alignment with optimal behavior is better explained by integrated value representations or by selective modulation of individual decision features.

**Table 1:** Overview of predictive variables representing behavioral models.

Variable name	Explanation	Range of possible values in the task	Example value of respective task variables depicted in Fig 2
Optimal policy (*Q*-value difference, or ΔQ-values)	Difference between state–action values computed with dynamic programming for all possible future states *s*.	−0.84–0.625	Cannot be depicted since it depends on the context (current state and time-point) of all relevant variables
multi-feature policy	Combination of multiple predictors influencing dynamic choice: • Binary energy state BES • Wait when safe WWS • Probability of gaining food p(gain) • Risk of threat encounter r(threat)	See description of the models regarding the individual predictors	See description of the models regarding the individual predictors
Marginal value model (based on marginal value theorem; MVT)	Expected gains are averaged across all previous trials and used as a cutoff. Model says foraging if current weather is above and wait if current weather is below average.	0 (wait) and 1 (forage)	Not available since marginal value depends on the average expected gain of previous trials
Expected gain	Probability of gaining food *p* multiplied by respective magnitude of foraging gain *g* plus *q* times −2 (loss when foraging unsuccessful) and *r* times −3 (loss when encountering a predator).	−2 to 0.5	−0.2 in the example without threat, ~−0.65 in the example with threat
Probability of gaining food (p(gain) with q=1−p)	Probability of gaining energy in the magnitude indicated on the screen. This is inferred by counting the number of food-containing fields in a weather grid.	0.2–0.67	0.6 in the example without threat, 0.67 in the example with threat
Risk of predator encounter (r(threat))	Probability of encountering a predator when deciding to forage.	0–0.67	0.33 in the example with threat
Weather type	In each forest, the grids representing weather types can be categorized into “good” weather with higher probability of foraging success and “bad” weather with lower probability of success.	Categorical: 0 “bad” or 1 “good”	0 for example with threat, 1 for example without threat (since corrected success probability is higher)
Binary energy state (BES)	Participants must forage in energy state 1. Therefore, this variable is binarized as 1 if the energy state is 1, and 0 for all the other energy states.	Binary: 1 or 0	0 (since waiting does not lead to starvation)
Wait when safe (WWS)	If energy state is higher than days left in a forest, participants can simply wait in order to win the game. Therefore, this variable is 0 if energy state is higher than days left, and 1 for all other energy states. The number of days in a forest was not displayed and had to be tracked by participants themselves.	Binary: 1 or 0	0 if only 3 days left, otherwise 1
Win-stay-lose-shift (WSLS)	Possible strategy that uses the outcome of the former trial to decide in the current trial. This policy chooses foraging if the energy state increased and waiting if it decreased in the former trial.	Categorical: 1 “energy state increased” or 0 “energy state decreased”	Not available since it depends on previous trial in the context.

Because participants contributed a large number of repeated decisions, all computational models were estimated using a hierarchical Bayesian framework. This approach pools information across participants while preserving individual differences, allowing group-level and subject-level effects to be estimated simultaneously. By leveraging the hierarchical structure of the data, hierarchical models improve statistical efficiency and stabilize parameter estimates, particularly in repeated-measures designs with many observations per participant. We first compared models across the full dataset using PSIS-LOO cross-validation to identify the best account of participants’ behavior (Fig 4A). The multi-feature policy provided the best predictive performance (ΔELPD = 0), substantially outperforming all alternative models, including the ΔQ-based model (ΔELPD = −465). This suggests that behavior is best captured by the combined influence of multiple task-relevant features, rather than any single decision variable. In contrast, models based on isolated features performed markedly worse (ΔELPDs > −1300), indicating that no single variable was sufficient to capture behavior. Importantly, this comparison does not imply that integrated value signals are irrelevant, but rather that their influence is not fully captured by any single model in isolation. To directly assess the joint contributions of feature-based and integrated value signals, we next fitted a unified hierarchical model including the components of the multi-feature model, ΔQ-values, and their interactions with condition (approach vs. avoidance; Fig 4B and 4C).

**Fig 4:**
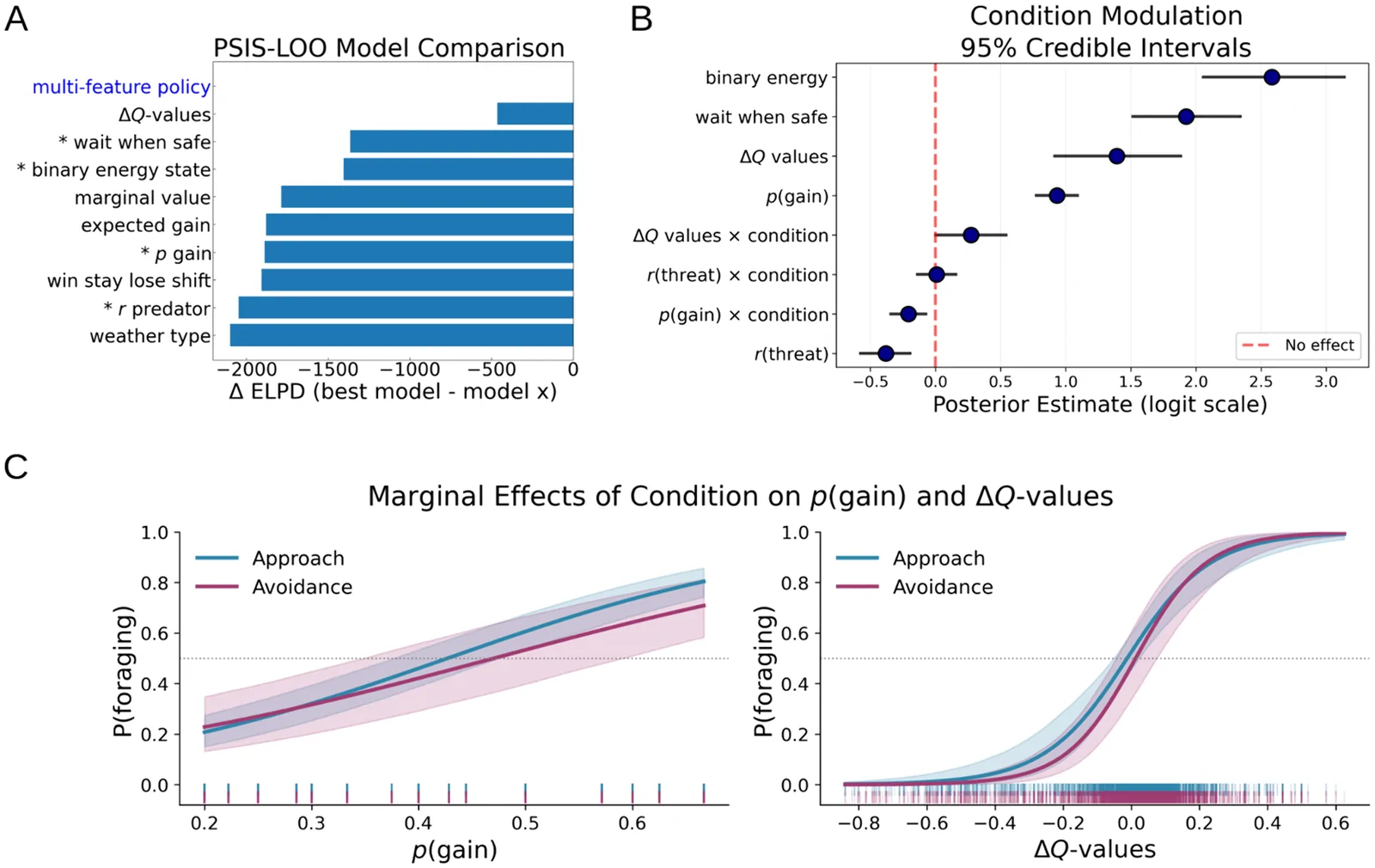
Model comparison and context-dependent feature reweighting. **(A)** PSIS-LOO model comparison across candidate models. Bars show differences in expected log predictive density (ΔELPD) relative to the best-performing model (multi-feature policy). The multi-feature policy outperformed all single-feature and alternative heuristic models, indicating that behavior is best captured by a combination of task-relevant features. Asterisks (*) denote features that were included in the multi-feature policy. **(B)** Posterior estimates (mean ± 95% credible intervals) from the unified hierarchical model showing main effects and condition-dependent modulation of decision features on foraging behavior (logit scale). Positive values indicate increased probability of foraging. Avoidance contexts selectively modulated feature weights, including reduced sensitivity to p(gain) and increased influence of ΔQ-values. A full overview of all model effects, including block modulation and feature interactions, is provided in S1 Fig. **(C)** Marginal effects of condition on p(gain) (left) and ΔQ-values (right). Lines show predicted probability of foraging (mean ± 95% credible intervals) for approach and avoidance conditions. Under avoidance, the influence of p(gain) is reduced, whereas sensitivity to ΔQ-values is increased, indicating stronger alignment with integrated value signals. Rug plots indicate the distribution of observed data.

At the level of main effects, behavior was shaped by multiple decision features. Participants were more likely to forage with increasing gain probability (p(gain): β=0.93, 95% CI [0.77, 1.10]) and increasing value differences (ΔQ: β=1.39, [0.90, 1.85]). In addition, two features exerted particularly strong influences: waiting when safe (β=1.92, [1.53, 2.33]) and binary energy state (β=2.59, [2.05, 3.14]), indicating that choices were strongly constrained under boundary conditions where action requirements become clear. Threat probability (r(threat)) showed a reliable negative effect (β=−0.39, [−0.58,−0.20]), reflecting reduced foraging as risk increased. These results indicate that choice behavior was influenced by multiple task-relevant features spanning reward, threat, and state-dependent boundary conditions.

Regarding policy modulation by condition, there was no evidence for a global shift in overall decision policy, as the main effect of condition was not reliably different from zero (β=−0.23, [−0.71,0.25]). This indicates that contextual differences in behavior cannot be explained by a uniform change in response tendencies, but instead reflect selective feature reweighting, that is, context-dependent changes in how decision-relevant features influence choice. Most notably, the effect of gain probability was significantly reduced in avoidance contexts (p(gain)× condition: β=−0.20, [−0.34,−0.07]), as also reflected in the attenuated slope relating gain probability to foraging probability (Fig 4C left). In contrast, threat-related modulation was weaker: the interaction between threat probability and condition did not differ reliably from zero (β=−0.02, [−0.15,0.12]).

Critically, condition also modulated sensitivity to the integrated decision variable ΔQ (ΔQ× condition: β=0.28, [0.00, 0.55]), with choices in avoidance contexts showing greater alignment with the integrated value structure of the task (Fig 4C right). This effect persisted both when ΔQ-values were included alongside reward-, threat-, and state-related predictors in the unified model and when ΔQ-values were residualized with respect to those predictors (S1 Fig B), indicating that increased alignment with ΔQ cannot be reduced to variance shared with the modeled features alone. Consistent with this interpretation, gain-threat interactions did not show reliable contributions when ΔQ-values were included, suggesting that their joint influence was largely captured by the integrated value signal.

The unified model additionally included interactions with block number to capture experience-dependent changes, as well as gain–threat interactions (p×r) and their modulation by condition (p×r× condition). Across blocks, participants showed systematic shifts in behavior, characterized by an overall reduction in foraging (β=−0.58, [−0.87,−0.30]) and increased alignment with optimal value differences (ΔQ× block: β=0.17, [0.03, 0.31]). Importantly, the increased alignment with the optimal policy across blocks was accompanied by systematic changes in the influence of multiple decision features, suggesting that experience-dependent adaptation is consistent with structured changes in feature integration rather than a simple global shift toward optimal responding. The influence of the WWS feature also increased over time (β=0.37, [0.09, 0.66]), while sensitivity to threat became slightly more negative (r(threat)×block: β=−0.10, [−0.16,−0.03]). Other feature–block interactions were not reliably different from zero. As these experience-dependent effects were not central to our primary question of context-dependent feature reweighting, they are not shown in detail in the main figure. Fig 4 summarizes the effects central to context-dependent feature reweighting, while a complete overview of all parameters, including block and gain–threat interaction terms, is provided in S1 Fig.

Taken together, these results indicate that behavior reflects the combined influence of multiple decision features rather than any single variable. Environmental context did not produce a global shift in overall response tendency, as reflected by the absence of a condition main effect, but instead selectively modulated the influence of specific features: avoidance contexts reduced the impact of reward-related information while increasing sensitivity to the integrated value structure of the task (ΔQ). Importantly, this increased alignment with ΔQ-values cannot be reduced to the combined effects of individual features, suggesting that behavior captures aspects of the integrated value signal beyond those represented by the modeled feature set. These findings are consistent with context-dependent changes in the weighting of decision features, rather than a uniform shift in decision policy. While the observed pattern is consistent with increased alignment to ΔQ-values, this effect appears to emerge through differential modulation of individual feature weights rather than a uniform change in decision policy.

To ensure that the compared models were sufficiently distinguishable and recoverable, we performed a model recovery analysis using synthetic datasets generated from each candidate model and refit with the full model space (Fig 5). Recovery accuracy was quantified as the proportion of simulations in which the generating model was correctly identified as the best-fitting model based on PSIS-LOO predictive performance. Overall, recovery was highly accurate across all candidate models, with correct classification rates ranging from 0.96 to 1.00. Misclassifications were rare and occurred primarily when simple single-feature models were occasionally recovered as the more flexible multi-feature policy. Importantly, the multi-feature policy and ΔQ-based model showed perfect recovery, indicating that the main theoretical distinction underlying the present study was highly separable. Although some recovery fits involving highly flexible models applied to data generated by simpler single-feature policies produced elevated $\hat{R}$ values or reduced effective sample sizes, overall model recovery performance remained highly accurate, suggesting that these diagnostic issues primarily reflected weak parameter identifiability in near-separable synthetic choice patterns rather than systematic model confusability. Together, these results suggest that the observed superiority of the multi-feature policy in the empirical data is unlikely to reflect model overlap or insufficient discriminability between candidate models.

**Fig 5:**
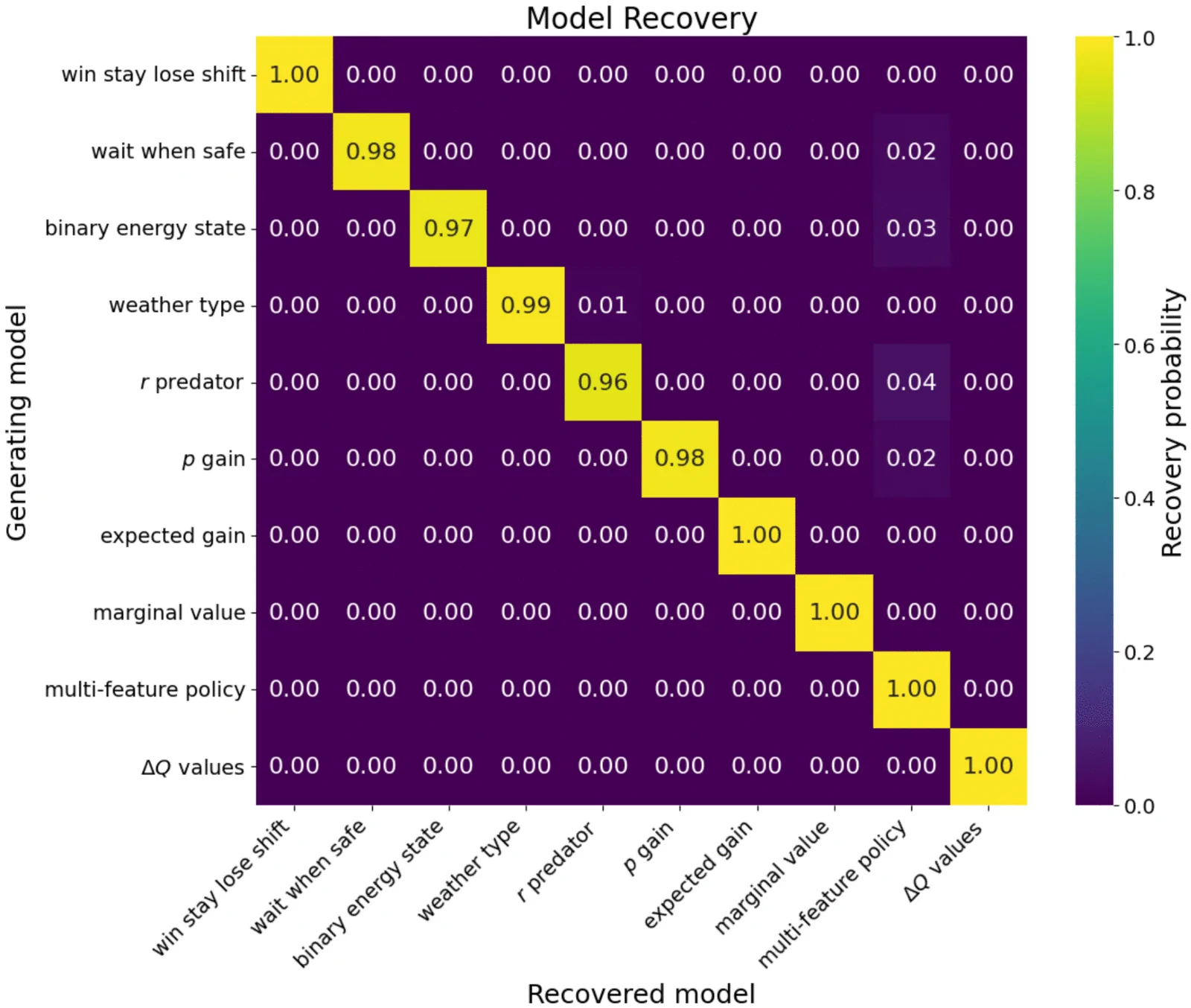
Model recovery analysis. Confusion matrix showing model recovery performance across all candidate policy models. Synthetic datasets were generated from each model using posterior parameter estimates from the empirically fitted hierarchical Bayesian models and subsequently refit using the full candidate model space. Recovery analyses used conditional simulation procedures in which choices were regenerated while preserving the original trial structure and task states. Values indicate the proportion of simulations in which a given generating model (rows) was correctly or incorrectly identified as the best-fitting model (columns) based on PSIS-LOO predictive performance. Recovery accuracy was high across all candidate models (0.96–1.00), with only minor misclassification of some simple single-feature models as the more flexible multi-feature policy. Importantly, the multi-feature policy and ΔQ-based model showed perfect recovery, indicating strong discriminability between the principal competing accounts examined in the study. Although some recovery fits involving highly flexible models applied to data generated by simpler single-feature policies produced elevated $\hat{R}$ values or reduced effective sample sizes, overall model discriminability remained robust.

## 3 Discussion

### 3.1 Behavior reflects context-dependent reweighting of decision features

The present study investigated how decision-making adapts in sequential approach–avoidance contexts by comparing value-based and feature-based accounts of behavior. Across conditions, choices were best explained by the combined influence of multiple task-relevant features rather than any single decision variable. Importantly, the overall superiority of the multi-feature policy indicates that behavior remained primarily influenced by multiple decision-relevant features across both contexts. Furthermore, environmental context did not induce a global shift in decision policy, as evidenced by the absence of a reliable main effect of condition on overall response tendencies. Instead, avoidance contexts selectively modulated the influence of specific decision features while also increasing alignment with the integrated value structure of the task (ΔQ). Together, these findings suggest that adaptive behavior arises through context-dependent changes in how decision-relevant information contributes to choice, rather than through a qualitatively distinct decision strategy or a uniform shift in response policy.

### 3.2 Avoidance contexts increase alignment with integrated value signals

A key finding was that choices in avoidance contexts showed greater alignment with the integrated value signal derived from the task structure. Even after accounting for feature-based predictors, ΔQ values explained additional variance in choice behavior, and the influence of ΔQ increased under avoidance. This effect persisted in models that explicitly accounted for reward, threat, and state-dependent boundary conditions (BES and WWS), as well as after residualization analyses, indicating that the observed condition effect was not fully captured by the modeled feature set. Importantly, the increased influence of ΔQ was not accompanied by longer response times, and comparable survival rates and ΔQ-value distributions across conditions argue against simple explanations based on increased deliberation, task difficulty, or environmental structure.

Together, these findings suggest that avoidance contexts altered how decision-relevant information contributed to choice, resulting in greater alignment with the integrated value structure of the task without evidence for a global shift in decision policy.

### 3.3 Implications for models of adaptive choice

The present findings have important implications for how adaptive behavior is conceptualized in sequential decision-making. Traditional accounts often frame adaptation as a shift between qualitatively distinct strategies, such as transitions between feature-based and integrated value-based policies or between model-free and model-based systems (e.g., [23,24]). In contrast, the current results suggest that adaptive behavior may instead emerge from continuous adjustments in how a shared set of decision-relevant features is weighted and combined.

A key finding was that avoidance contexts increased alignment with the integrated value signal derived from the task structure (ΔQ), while simultaneously reducing the influence of reward probability and selectively modulating other decision features. One possible explanation for this pattern is that avoidance environments place greater demands on coordinating reward and threat information. Although higher reward opportunities were associated with greater threat in both conditions, avoidance environments were structured such that avoiding the higher-threat option was more often advantageous under the optimal policy. Under these circumstances, reward probability alone becomes a less reliable guide to action selection, potentially increasing the importance of integrating multiple decision-relevant features.

Importantly, the influence of ΔQ explained behavior beyond the individual feature predictors. However, this does not imply that participants explicitly computed ΔQ-values or switched to a qualitatively distinct decision strategy. Rather than representing competing process-level accounts, feature-based and integrated value-based models may capture different levels of description. Feature-based models characterize the information influencing choice, whereas integrated value models characterize how that information is combined within the structure of the task. Consistent with optimization-based perspectives (e.g., [15]), complex or near-optimal behavior may emerge through the tuning of relatively simple parameterized policies, whose lower-dimensional structure provides a scalable and flexible means of adaptation without requiring explicit construction of exhaustive value representations. From this perspective, greater alignment with integrated value signals may arise through adaptive changes in how decision-relevant features are weighted and combined.

This interpretation is consistent with proposals that value signals reflect emergent summaries of underlying computations rather than distinct decision processes (e.g., [5]). It is also compatible with resource-rational accounts of behavior under computational constraints (e.g., [10]), because behavioral flexibility can be achieved by adjusting a limited set of feature weights rather than computing new policies from scratch. More generally, the results suggest that flexible behavior can emerge not only through switching between internal models of the environment [25], but also through gradual changes in how the same decision-relevant information is weighted across contexts. Future work should investigate the neural and computational mechanisms underlying these adjustments, as well as their generality across different task structures and populations.

### 3.4 Limitations

First, the sample comprised 29 participants. Although each participant contributed a large number of repeated decisions and all models were estimated hierarchically, larger samples will be needed to establish the robustness and generalizability of the present findings. Second, the manipulation of threat was purely symbolic and not validated with physiological or subjective measures, leaving open whether the avoidance condition induced genuine affective responses or instead reflected differences in attention or task demands. Furthermore, the task remains an abstract and simplified environment, and it is unclear how well the observed patterns generalize to real-world decision-making involving more salient or consequential threats. Nevertheless, predator encounters imposed substantially greater immediate survival costs than starvation within the task structure, thereby creating an ecologically motivated conflict between reward-related and threat-related outcomes. Third, the optimal policy serves as a normative benchmark rather than a process-level account; thus, increased alignment with ΔQ-values should not be interpreted as evidence that participants explicitly compute or implement this policy, but may instead reflect improved integration of decision-relevant features or reduced decision noise.

Importantly, although alternative explanations for the ΔQ-values × condition interaction, such as differences in task difficulty or cognitive demand, cannot be fully excluded, several analyses argue against a simple “easier avoidance condition” account. In particular, ΔQ-value distributions were highly similar in shape across conditions, although a small but statistically significant difference was detected, indicating subtle distributional differences rather than a clear shift in task difficulty. Moreover, response times did not differ as a function of decision difficulty or context, and the increased influence of ΔQ under avoidance persisted even when ΔQ values were residualized with respect to the multi-feature model predictors. Notably, an exploratory policy-arbitration analysis (**S6 Fig**) produced a similar pattern of results while explicitly accounting for overlap between competing policy representations, suggesting that the observed increase in ΔQ-alignment cannot be readily explained by representational overlap alone. Nevertheless, the present approach does not fully resolve the relationship between feature reweighting and integrated value representations, and further work will be required to more directly test these alternatives.

Finally, while we accounted for block-related changes, the present framework does not fully dissociate the computational mechanisms underlying these effects. In particular, the observed ΔQ-values × block interaction indicates that behavior changed systematically across the experiment, but the current modeling approach remains descriptive and does not specify whether these changes reflect learning, strategic adaptation, fatigue, attentional shifts, or other latent processes. Thus, we do not provide a generative account of how context-dependent feature reweighting emerges over time. Future work should therefore combine larger samples with process-level computational models that more directly link behavioral adaptation to underlying cognitive mechanisms, including through targeted manipulations of task structure, duration, or affective salience.

## 4 Methods

### 4.1 Ethics statement

Ethics approval was given by the local ethics committee of the medical faculty of Heidelberg University. All participants provided written informed consent prior to participation.

### 4.2 Participants

We tested 29 healthy participants (15 female, 14 male; mean age = 23.93 years, SD = 3.73) in the lab, who were recruited via fliers and online tenders. Participants were asked whether they had any history of psychiatric or neurological diagnoses, which was an exclusion criterion. None of the invited subjects were excluded.

### 4.3 Description of the hunter-gatherer task with predators

Our task (see Fig 2) was adapted from the “Hunter-Gatherer Task” by Korn and Bach [21]. The task consisted of multiple mini-blocks (“forests”), each containing two probabilistic environments (“weather types”) with varying chances of gains and losses. Participants’ goal was to “survive” by preventing their discrete energy levels (life points) from reaching zero over a series of trials (“days”) within each forest, with a monetary reward for survival. On each trial, one weather type was randomly selected, indicating the probability and magnitude of food gains. Participants chose to either forage (hunt/gather) or wait for better conditions. Probabilities of food gain were visually represented as colored dots on a grid. Waiting incurred a small, guaranteed energy loss (1 point), while unsuccessful foraging resulted in a larger loss (2 points), creating a trade-off.

Building on [21,22], we introduced predators as an additional probabilistic threat, displayed as roaming red frames on the grid. If a participant foraged and encountered a predator, they lost even more energy (3 points), simulating the increased cost of a fight-or-flight reaction. Threat-related outcomes were implemented symbolically through probabilistic predator encounters rather than direct aversive stimulation. Within the task structure, predator encounters imposed substantially greater immediate survival costs than starvation, thereby creating a sequential trade-off between reward acquisition and threat avoidance. Importantly, in every forest, the weather type with a higher probability of finding food also had a higher predator risk, creating an approach–avoidance conflict. However, the balance between these competing factors differed across forests. Based on the optimal policy derived from the task structure (described in **4.4 Computation of the Optimal Policy**), some forests favored approaching the higher-reward, higher-threat option, whereas others favored avoiding it. These environments formed the approach and avoidance conditions used throughout the experiment.

Each weather type was represented by a grid with 2–10 fields, each equally likely to be the participant’s landing location after foraging. The probability of finding food (*p*) was defined by the ratio of food-containing fields to total fields, ranging from 0.2 to 0.67 to avoid near-zero chances. Similarly, predator risk (*r*) was defined as the probability that both participant and predator landed on the same field, ranging from 0 to 0.67 (2 predators and 3 fields), based on the number of predators relative to total fields. Foraging failure probability (*q*) was 1−p.

Participants completed 72 forests divided into four blocks of 18. Each forest lasted 8 days, with one of the two weather types randomly selected on each day (50% probability). The task included 36 approach forests and 36 avoidance forests. Forests differed in the configuration of food availability, predator risk, and grid size, thereby generating a diverse set of sequential decision environments while preserving the overall task structure.

Participants began each forest with an energy level randomly between 4 and 5, capped at a maximum of 6 discrete points, creating 7 possible energy states (0–6). Coupled with two weather types, this yielded 14 possible states per forest. Falling to zero energy ended the forest prematurely without reward. This setup satisfies the criteria for a fully observable Markov Decision Process (MDP), enabling computation of optimal state–action values through dynamic programming. The resulting optimal policy prescribes the action with the highest expected long-term value in each state. To ensure meaningful decision trade-offs, forests in which the optimal policy showed little preference between weather types were excluded. Specifically, forests with an absolute difference in foraging preference below 10 across states and time-points between the two weather types were removed.

### 4.4 Computation of the optimal policy

The experimental setup allowed to derive the optimal policy from a fully observable MDP. Since the transition probabilities between states were fully overt, the state–action values could be computed iteratively via dynamic programming (or backward induction). Fig 1 illustrates the requirements (state–action transitions and transition matrix) for computing the optimal policy with the MDP algorithm.

Backward induction was performed by iteratively computing state–action values for all states and time points according to the Bellman framework:


$$\begin{array}{c} Q^{\pi }(s,a)=E_{\pi }\left[G_{t}|S_{t}=s,\;A_{t}=a\right]=E_{\pi }\left[\sum_{j=0}^{T}\gamma^{j}R_{t+j+1}|S_{t}=s,\;A_{t}=a\right], \end{array}$$ (1)

where 𝔼 is the expected value of the total return *G* when starting from state *s* at time *t* and following policy π. We only considered the discount factor γ = 1, since *t*he game mechanics of our task did not necessitate nor motivate discounted rewards over time. The policy π can, thus, be computed from the argmax of the average future rewards of the action set *a* as follows:


$$\begin{array}{c} \pi (s)=*{arg\;max}_{a}\sum_{s^{\prime },r}p(s^{\prime },r\;|\;s,a)\left[r+\gamma V(s^{\prime })\right]. \end{array}$$ (2)

Accordingly, the action values for foraging $Q_{f}$ and waiting $Q_{w}$ can be transformed into a single state–action value difference (ΔQ-value) in the following manner:


$$\begin{array}{c} \Delta Q=Q_{forage}-Q_{wait}. \end{array}$$ (3)

If ΔQ is positive, the optimal policy suggests foraging. If ΔQ is negative, the optimal policy suggests waiting. If ΔQ is zero, the optimal policy is indifferent. Rather than fitting the deterministic optimal policy directly, ΔQ-values were used as continuous predictors within a logistic choice model. This allows graded choice probabilities to be modeled as a function of the strength of the value difference between actions, thereby accommodating uncertainty and variability in human decision-making. The code for calculating ΔQ was made publicly available on GitHub (see https://github.com/SAEG64/fora02/tree/main).

### 4.5 Task presentation

At the beginning of a forest, participants were shown both weather types in the so-called forest phase (3.5 s), followed by 8 consecutive decision and feedback phases (the so-called “days”) until completion of a forest. The feedback was displayed for 2 s. Before each decision phase, a random fixation time between [0.5, 3.8] appeared in the form of a blank screen. Participants had to give a response within 3 s, otherwise the option ‘waiting’ was chosen by default. In case of no response, these decisions were excluded from the data when conducting model comparisons. Fig 2 illustrates the details of the task presentation. Before doing the task, subjects received written instructions about the setup. The full instructions sheet can be found in S1 Text.

### 4.6 Behavioral modeling

The main objective of this experiment was to examine how participants integrate reward and threat information when making sequential foraging decisions across different contexts. Rather than assuming a strict dichotomy between optimal (MDP-based) and feature-based strategies, our approach tests the extent to which behavior can be explained by a limited set of decision features and how their weighting varies across conditions. To this end, we compared a set of models ranging from single- to multi-feature models, as well as the state–action value difference derived from the optimal policy (ΔQ-values), which represents a normative benchmark for the best possible choice pattern in our task. A detailed description of all models is provided in Table 1.

Our task design includes several variables that can be directly derived from the task and can serve as simple feature policies: probability of gaining food, risk of threat encounter, weather type (good and bad), the internal (binary) energy state and the wait when safe policy (if sufficient energy points are collected, participants can simply wait to win the game). We also included the win-stay-lose-shift policy, which binarizes the evaluation of the last event in the choice history. These models represent directly observable and univariate features of our task, which do not require any further feature engineering. In addition to this, we included what we call “intermediate” models, a set of policies that require some recombination of variables to represent slightly more complex decision rules. Examples of such models include the expected energy gain model, which computes the weighted average of possible outcomes ($E[X]=\sum_{i}x_{i}p_{i}$; where $x_{i}$ denotes the magnitude of energy gain or loss and $p_{i}$ the corresponding probability), and a marginal value model derived from this quantity, in which foraging is chosen when the current expected gain exceeds a running average, and waiting otherwise. The marginal value model stems from the marginal value theorem (MVT), in which patch foraging behavior is explained with a threshold value computed from the mean expectancy of sequentially experienced environments (see [26,27]).

We also constructed a multi-feature policy that captures behavior using a small set of key task variables: reward and threat probabilities (*p* and *r*), as well as two indicators for situations in which the decision is effectively straightforward (binary energy state, BES; and “wait when safe,” WWS). Instead of treating these as separate features, the model combines them to predict choices. This allows it to approximate optimal decisions in simple situations, while relying on a balance of reward and threat information when trade-offs are present. In this way, the model can be used to test how the importance of these features changes across states and task conditions. Although the multi-feature model includes multiple predictors, parameter estimation was regularized through hierarchical shrinkage, ensuring that improved predictive performance reflects generalizable structure rather than overfitting.

The optimal policy (ΔQ-values) is based on a single combined measure of the decision, whereas the multi-feature policy uses several task-relevant features together to predict choices. This gives the multi-feature model more flexibility in how it captures behavior. However, all of these features directly reflect central aspects of the task rather than introducing additional assumptions. To ensure a fair comparison, we evaluated models based on how well they predict new data, so that better performance reflects more accurate predictions rather than simply greater flexibility.

### 4.7 Model comparison

All models were fit using hierarchical Bayesian logistic regression using the Python package PyMC, allowing parameters to vary across participants while being constrained by group-level distributions (partial pooling). This approach improves parameter estimation by sharing statistical strength across individuals while preserving meaningful between-subject variability. Group-level regression coefficients were assigned weakly informative normal priors centered at zero (*N*(0,1)). Variance parameters governing subject-level variability were assigned Exp(1) priors. Subject-level effects were estimated hierarchically using non-centered parameterization to improve sampling efficiency and stabilize No-U-Turn Sampler (NUTS) estimation. Posterior inference was performed using Hamiltonian Monte Carlo with the NUTS sampler implemented in PyMC. Unless otherwise specified, models were estimated using 4 independent Markov chains with 1000 tuning iterations and 1000 posterior sampling iterations per chain (target_accept = 0.90). All continuous predictors were z-scored prior to model fitting to facilitate parameter estimation, comparability across features, and the use of weakly informative priors on a common scale. For the multi-feature policy model, only continuous predictors (p(gain) and r(threat)) were standardized, while binary predictors (“wait when safe” and “binary energy state”) remained unscaled. The multi-feature model additionally included fixed pairwise interaction terms between predictors, while retaining random slopes for the corresponding main effects.

For each model, choices were predicted from the corresponding decision variable (or set of predictors, in the case of the multi-feature policy) using a logistic link function:


$$\begin{array}{c} \eta_{i,t}=\mathrm{logit}\;\left[P({forage}_{i,t})\right]=\beta_{0,i}+\sum_{k}\beta_{k,i}X_{k,t}. \end{array}$$ (4)

where $X_{k,t}$ denotes the value of predictor *k* on trial *t*, and $\beta_{k,i}$ the corresponding subject-specific weight for participant *i*. The intercept $\beta_{0,i}$ captures each subject’s baseline tendency to choose the foraging option. The resulting linear predictor is mapped onto choice probabilities via the logistic link function:


$$\begin{array}{c} P\left({forage}_{i,t}\right)=\frac{1}{1+\exp\left(-\eta_{i,t}\right)}. \end{array}$$ (5)

All models showed satisfactory convergence diagnostics (all $\hat{R}$ < 1.01, no divergent transitions, and effective sample sizes within recommended ranges). Posterior predictive checks further indicated that the fitted models reproduced the main patterns observed in the behavioral data (S5 Fig). Models were compared using Pareto-smoothed importance sampling leave-one-out cross-validation (PSIS-LOO), which estimates out-of-sample predictive performance by evaluating how well each model generalizes to unseen data. Model performance was quantified using the expected log predictive density (ELPD), with higher values indicating better predictive performance. Differences in ELPD (ΔELPD) were used to assess the relative support for competing models.

We assessed the similarity between model predictors and found that correlations between models remained moderate (all r≤0.7; see S2 Fig). These levels are below typical thresholds for problematic multicollinearity. Furthermore, we ensured the validity of our model comparison by performing both model and parameter recovery analyses, following established guidelines [28,29]. Synthetic datasets were generated from posterior parameter estimates of the empirically fitted hierarchical models while preserving the original trial structure and task states (conditional simulation procedure), and subsequently refit using the full candidate model space. Recovery analyses used the same hierarchical Bayesian logistic regression framework as the empirical analyses, including non-centered parameterization, weakly informative priors, and partial pooling across participants. For the multi-feature policy, all pairwise interactions between predictors were retained except for the interaction between “wait when safe” and “binary energy state,” which was excluded because the two variables represented mutually exclusive boundary-state conditions by task design. Including this interaction introduced structurally redundant parameterization that reduced parameter recoverability without improving model expressiveness. Recovery fits were estimated using 4 Markov chains with 500 tuning and 500 posterior sampling iterations per chain (target_accept = 0.95). Model recovery results are reported in Fig 5 (see **Results**, **2.2 Model Inference**), while parameter recovery is provided in the Supplementary Materials (S3 and S4 Figs). Overall, these analyses demonstrated sufficient model discriminability and parameter recoverability for meaningful model comparison. The full codebase for all modeling analyses is publicly available (see https://github.com/SAEG64/fora02/tree/main).

### 4.8 Control analyses: Task difficulty and response times

To assess whether task difficulty differed across conditions, we quantified decision difficulty using the absolute optimal policy value difference (|ΔQ|) across all state–action pairs of the MDP and compared the resulting distributions between approach and avoidance conditions using a Kolmogorov–Smirnov test.

To examine whether decision difficulty influenced response times, we tested whether |ΔQ| predicted log-transformed response times. In addition, we examined effects of condition (approach vs. avoidance) and their interaction using linear regression.

## Supporting information

S1 Text Instructions provided to participants for the Hunter/Gatherer Game. (PDF)

S1 FigFull hierarchical moderation model: main effects and interaction terms.Posterior mean estimates (points) and 95% credible intervals (horizontal lines) are shown for all main effects and interaction terms from the hierarchical logistic regression model. Estimates are reported on the logit scale; positive values indicate an increased probability of choosing the foraging option, whereas negative values indicate a decreased probability. The vertical dashed line denotes the null effect (0). **(A)** Model including raw ΔQ. **(B)** Model including ΔQ residualized with respect to the variables of the multi-feature model. Main effects reflect baseline contributions of decision variables, including reward probability (p(gain)), threat probability (r(threat)), model-derived optimal values (ΔQ), and state-dependent features (binary energy, wait when safe). Interaction terms capture how these contributions are modulated by experimental condition (approach vs. avoidance) and block number. Across both specifications, results indicate selective feature reweighting rather than global shifts (no condition main effect). In avoidance contexts, the influence of p(gain) is reduced, whereas alignment with ΔQ increases, while sensitivity to r(threat) remains comparatively stable. Block-related interactions indicate gradual changes in feature weighting over time, characterized by increasing alignment with ΔQ and boundary states (binary energy state and wait when safe). Crucially, the residualized model in **(B)** confirms that the condition-dependent increase in ΔQ persists beyond variance shared with other predictors, indicating a unique contribution of integrated value representations. Higher-order interactions, including p×r and p×r × condition, were not credibly different from zero in either model, suggesting that explicit feature interactions do not explain behavior beyond integrated value signals captured by ΔQ. Continuous predictors were standardized prior to model fitting. Condition was coded as 0 = approach and 1 = avoidance.(PDF)

S2 FigCorrelation structure of model features across all trials and trade-off trials.Pairwise Pearson correlations between optimal policy values (ΔQ) and candidate decision variables. Color intensity indicates correlation strength (−1 to 1). **(A)** Correlations across all trials. ΔQ-values show relatively weak associations with individual environmental features (e.g., p(gain): *r* = 0.05; r(predator): r=−0.15), but stronger correlations with state-dependent variables, particularly wait when safe (*r* = 0.70) and binary energy state (*r* = 0.37). Several heuristic and value-based predictors are more strongly intercorrelated (e.g., marginal value and expected gain: *r* = 0.68), reflecting shared structure among simpler decision variables. **(B)** Correlations restricted to trade-off trials (|ΔQ| near zero). In these difficult decision states, associations between ΔQ and value-based predictors increase (e.g., expected gain: *r* = 0.53; marginal value: *r* = 0.50), while correlations with state-dependent features are substantially reduced or absent. The multi-feature policy was not included, as it is a composite function of the predictors and therefore shares variance with them by construction. Correlations between such composite outputs and their constituent features are not informative for assessing multicollinearity among predictors. Accordingly, this analysis is intended as a descriptive check for dependencies among candidate features and does not replace formal model comparison or model recovery procedures. Overall, the pattern indicates that correlations observed across all trials are partly driven by extreme (easy) states, whereas in trade-off trials, predictors align more strongly with value-based representations, consistent with increased alignment with integrated value signals in difficult decisions.(PDF)

S3 FigParameter recovery results for the hierarchical Bayesian single-feature policy models.For each candidate model, 100 synthetic datasets were generated from posterior parameter estimates of the empirically fitted models and subsequently refit using the corresponding hierarchical model specification. For clarity and readability, the figure displays representative single-feature models spanning the principal classes of candidate policies; recovery performance for the remaining models showed comparable overall patterns. Recovery analyses used conditional simulation procedures in which choices were regenerated from the fitted models while preserving the original trial structure, task states, and stimulus configurations of the empirical dataset. Scatterplots depict the relationship between true generating parameters and recovered posterior mean estimates across simulations. Columns show recovery of the group mean intercept parameters (mean α), group-level intercept variability (SD α), group mean slope parameters / feature weights (mean β), and group-level slope variability parameters (SD β). The dashed red line indicates the identity line corresponding to perfect recovery, while the solid black line shows the fitted regression line with 95% confidence intervals. Recovery performance was quantified using regression slopes and Pearson correlations between true and recovered parameter values. Across models, parameter recoveries demonstrated generally good calibration and moderate-to-strong correlations, indicating reliable recovery of both central tendency and inter-individual variability parameters. Recovery analyses used the same hierarchical Bayesian logistic regression framework as the empirical analyses, including non-centered parameterization, weakly informative priors, and partial pooling across participants. Recovery fits were estimated using 4 Markov chains with 500 tuning and 500 posterior sampling iterations per chain (target_accept = 0.95). Convergence diagnostics produced frequent $\hat{R}$ and effective sample size warnings for datasets generated by very simple single-feature policies, particularly when these datasets were fit using more flexible hierarchical models. This likely reflects weak parameter identifiability and quasi-separable synthetic choice patterns arising from highly deterministic low-dimensional policies rather than failures of overall model recovery. Despite these warnings, the recovery analyses showed robust model discriminability and reliable recovery of the principal group-level parameters.(PDF)

S4 FigParameter recovery results for the hierarchical Bayesian multi-feature policy model.For each simulation, synthetic datasets were generated from posterior parameter estimates of the empirically fitted model and subsequently refit using the same hierarchical model specification. Recovery analyses used conditional simulation procedures in which choices were regenerated from the fitted policy while preserving the original trial structure, task states, and stimulus configurations of the empirical dataset. Scatterplots depict the relationship between true generating parameters and recovered posterior mean estimates across simulations. Panels show recovery of the group mean intercept parameters (mean α), group-level intercept variability (SD α), group mean slope parameters / feature weights (mean β), and corresponding group-level variability parameters (SD β) for the main effects and interaction terms included in the multi-feature policy model. The interaction between “wait when safe” (WWS) and “binary energy state” (BES) was excluded from the model because these predictors represented mutually exclusive boundary-state conditions by task design, and inclusion of the interaction introduced structurally redundant parameterization that reduced parameter recoverability without improving model expressiveness. Interaction coefficients are denoted using × between interacting predictors. The dashed red line indicates the identity line corresponding to perfect recovery, while the solid black line shows the fitted regression line with 95% confidence intervals. Recovery performance was quantified using linear regression slopes and Pearson correlations between true generating and recovered parameter estimates. Across parameters, recoveries demonstrated generally good calibration and moderate-to-strong correlations, indicating reliable identifiability of the principal feature weights and interaction effects of the multi-feature policy. Recovery analyses used the same hierarchical Bayesian logistic regression framework as the empirical analyses, including non-centered parameterization, weakly informative priors, partial pooling across participants, and all interaction terms specified in the fitted model. Recovery fits were estimated using 4 Markov chains with 500 tuning and 500 posterior sampling iterations per chain (target_accept = 0.95). As in the single-feature recovery analyses, convergence diagnostics occasionally produced elevated $\hat{R}$ values and reduced effective sample sizes, particularly for highly deterministic simulated datasets and interaction parameters. However, these issues did not materially affect overall parameter recoverability or model discriminability.(PDF)

S5 FigPosterior predictive checks for the primary inferential models.Posterior predictive distributions for **(A)** the multi-feature policy model and **(B)** the ΔQ-value model. Left panels show predicted choice probabilities for observed forage and wait decisions across all trials. Right panels show the corresponding distributions of predicted probabilities conditional on the observed behavioral choice. Both models reproduced the overall structure of participant behavior, with observed forage decisions generally associated with higher predicted foraging probabilities and observed wait decisions associated with lower predicted foraging probabilities. The multi-feature policy model showed stronger probabilistic separation between observed choice categories, consistent with its superior predictive performance in PSIS-LOO model comparison.(PDF)

S6 FigExploratory hierarchical policy-arbitration analysis examining continuous alignment with the multi-feature (MF) policy versus integrated ΔQ-value representations across individuals and conditions.A hierarchical Bayesian continuous mixture model was used to estimate trial-wise arbitration weights ($\pi_{t}$) reflecting the relative contribution of the MF versus ΔQ-based policy representations to observed choices. At the trial level, action probabilities were modeled as a probabilistic mixture of the two policy predictions: $P(a_{t})=\pi_{t}P_{MF}(a_{t})+(1-\pi_{t})P_{\Delta Q}(a_{t})$, where $P_{MF}(a_{t})$ and $P_{\Delta Q}(a_{t})$ denote the trial-wise action probabilities predicted by the MF and ΔQ-based models, respectively, and $\pi_{t}$ represents the arbitration weight favoring the MF policy. Importantly, the present analysis was designed to examine policy-level arbitration rather than feature-level modulation. Accordingly, both policy representations were first estimated independently using the hierarchical models from the primary analyses, and the resulting posterior action probabilities were then treated as fixed policy predictions within the arbitration model. This approach allowed arbitration tendencies to be examined while treating the independently estimated policy predictions as fixed inputs to the arbitration model. The ΔQ-based policy representation was operationalized using posterior predictions derived from the fitted Markov Decision Process (MDP) model based on integrated state–action value differences (ΔQ). The MF policy representation was operationalized using posterior predictions derived from the fitted hierarchical multi-feature model integrating reward probability (p(gain)), threat probability (r(threat)), and the boundary-condition features binary energy state (BES) and wait when safe (WWS). Arbitration weights were estimated hierarchically across participants and task conditions (approach vs. avoidance) while additionally controlling for block number and trial-wise policy similarity. Trial-wise policy similarity was quantified using the absolute difference between the MF and ΔQ-based policy predictions ($\Delta_{t}=|P_{\Delta Q}-P_{MF}|$), thereby accounting for trial-wise variation in overlap between the competing policy accounts. Importantly, policy-similarity distributions were highly comparable across conditions, indicating that the observed condition-dependent arbitration effects were unlikely to be explained solely by differences in overlap between the candidate policy representations across approach and avoidance environments. **(A)** Distribution of baseline arbitration weights across participants. Most participants showed greater overall alignment with the MF policy representation, consistent with the primary model-comparison analyses reported in the main text. However, arbitration weights formed a continuous distribution without evidence for sharply separable strategy classes, suggesting graded inter-individual variability in relative policy alignment. **(B)** Posterior probabilities of condition-dependent shifts toward increased ΔQ-based policy alignment in avoidance contexts. Most participants showed posterior probabilities substantially above 0.5, indicating a consistent directional tendency toward greater alignment with integrated-value representations under avoidance conditions despite substantial overlap between the MF and ΔQ-based policy predictions.(PDF)
