# Supplementary material for: Context-dependent feature modulation shapes human decision policies in approach–avoidance conflicts
Source: PLoS Comput Biol. 2026 Sep 30;22(9):e1014793. doi: 10.1371/journal.pcbi.1014793 (PMC13652344; doi:10.1371/journal.pcbi.1014793)
Supplement: Supplementary file 1 [file pcbi.1014793.s001.pdf]

S1 Text.

Hunter/Gatherer Game

Imagine the following situation: You are a hunter/gatherer and live in a world without grocery stores or supermarkets. You have to rely on yourself and try to get food and ultimately survive.

The following game principles apply:

- The aim of the game is to survive.
- You need food to survive. To prevent starvation, you have the option of hunting/gathering food or waiting every day.
- If you decide to wait, you will slowly lose the energy you need to survive.
- The foods you can hunt or gather have different nutritional values and can provide you with different amounts of energy.
- However, hunting and gathering is subject to a certain degree of uncertainty. This means that your hunt/search can also be unsuccessful, in which case you will even lose additional energy points on the day.
- The chances of a successful hunt or search depend on the weather. If the weather is good, you are more likely to find food.
- You now have to decide time and again whether to take the risk of hunting/searching under the given weather conditions or whether you would rather wait a day and hope for better weather conditions.
- If you have collected enough food to survive until the end of the current game, then all you can do is wait. There is no extra reward for collecting more food. The only thing that counts is whether or not you can keep your supplies above 0 within a certain period of time.
- Should you decide to hunt/search, it is possible that you will encounter one or more predators. During this encounter, you will have to fight or flee, which will consume a lot of energy. This strain can push you to your limits or even lead to your death.
- You have a kind of sensor that tells you whether and how many predators are moving in your vicinity under the given weather conditions.

Specification of the game

- In the game, your energy balance is displayed in the form of a bar. You can have a maximum of 6 energy/life points.

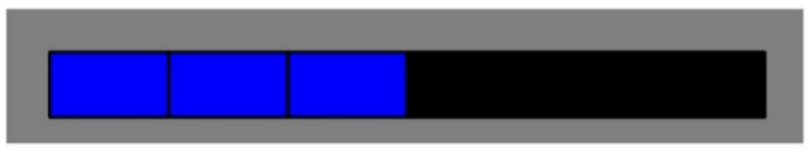

- You spend 8 days in each forest (8 passes). After that, you enter a new forest. The aim is to survive 8 days in a row in the same forest by keeping your life points above 0. However, if you cannot find any food and reach 0 life points, you are dead and automatically move on to the next forest.
- At the beginning of each new forest, you start with 4 or 5 life points at random.
- In each forest there are two different conditions (so-called weather types) that can occur randomly on each of the 8 days. These are introduced at the start of the round.
- The two weather conditions each consist of 2 to 10 white fields, the number of which is chosen at random. Each field contains either a food source (green dots) or none (red dots).

Example forest 1

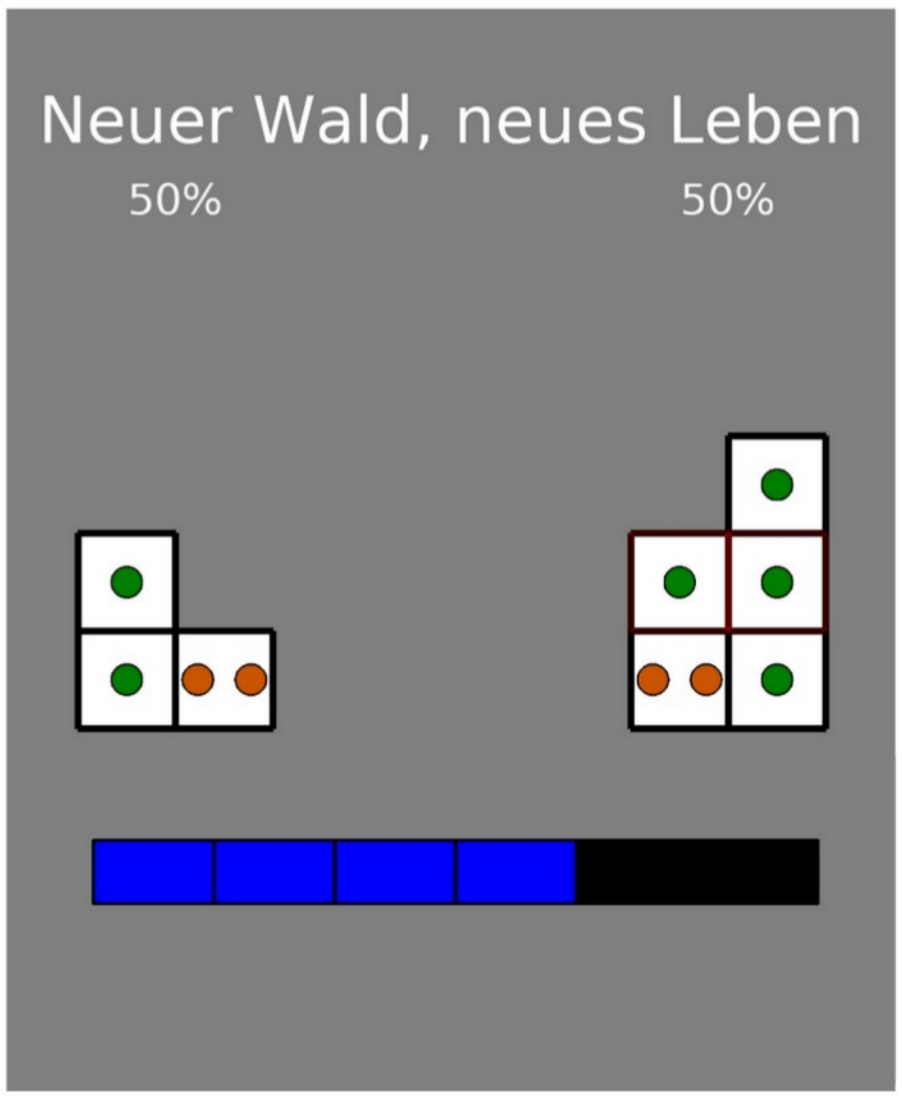

Example forest 2

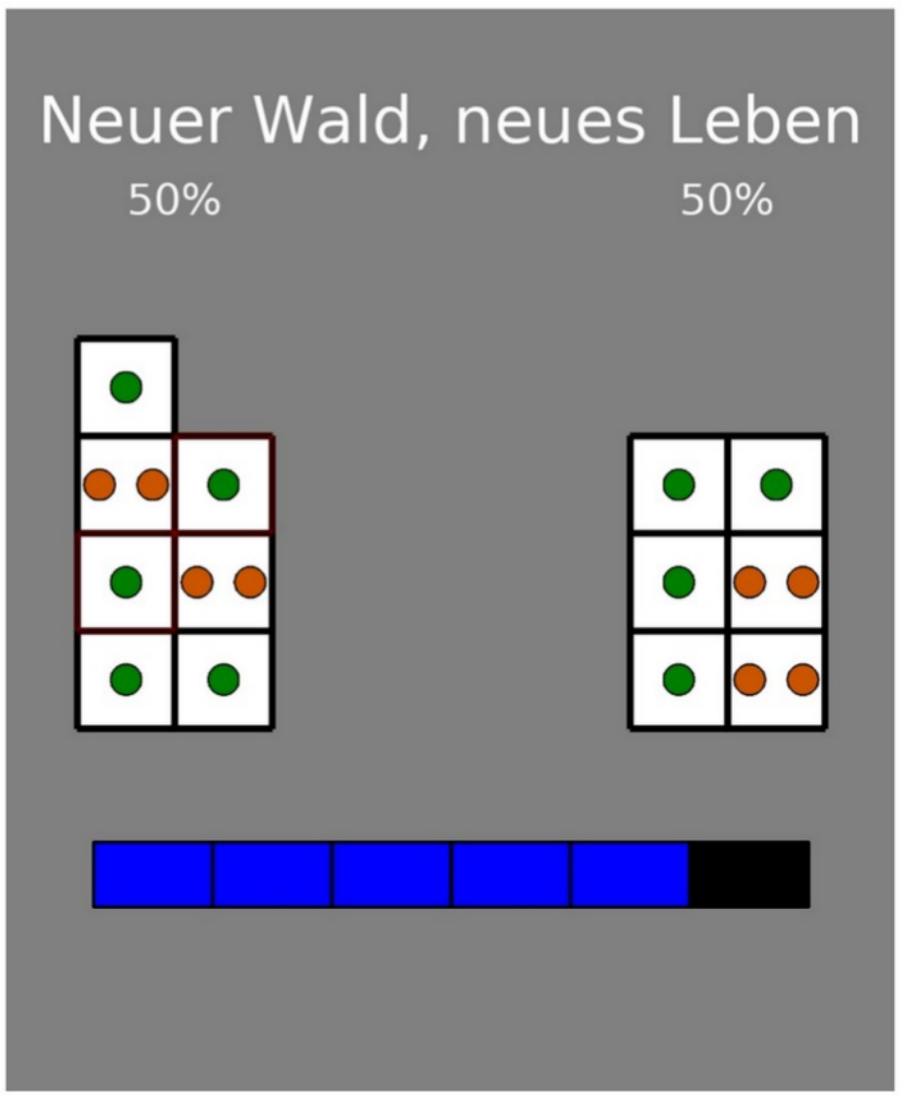

- One of the weather conditions is presented every day. By clicking the arrow key in the direction of the weather, you decide to go hunting/searching.
- If you click in the other direction using the arrow key, decide against the hunt/search and wait until the next day.

Example 1, Day 1: Choices

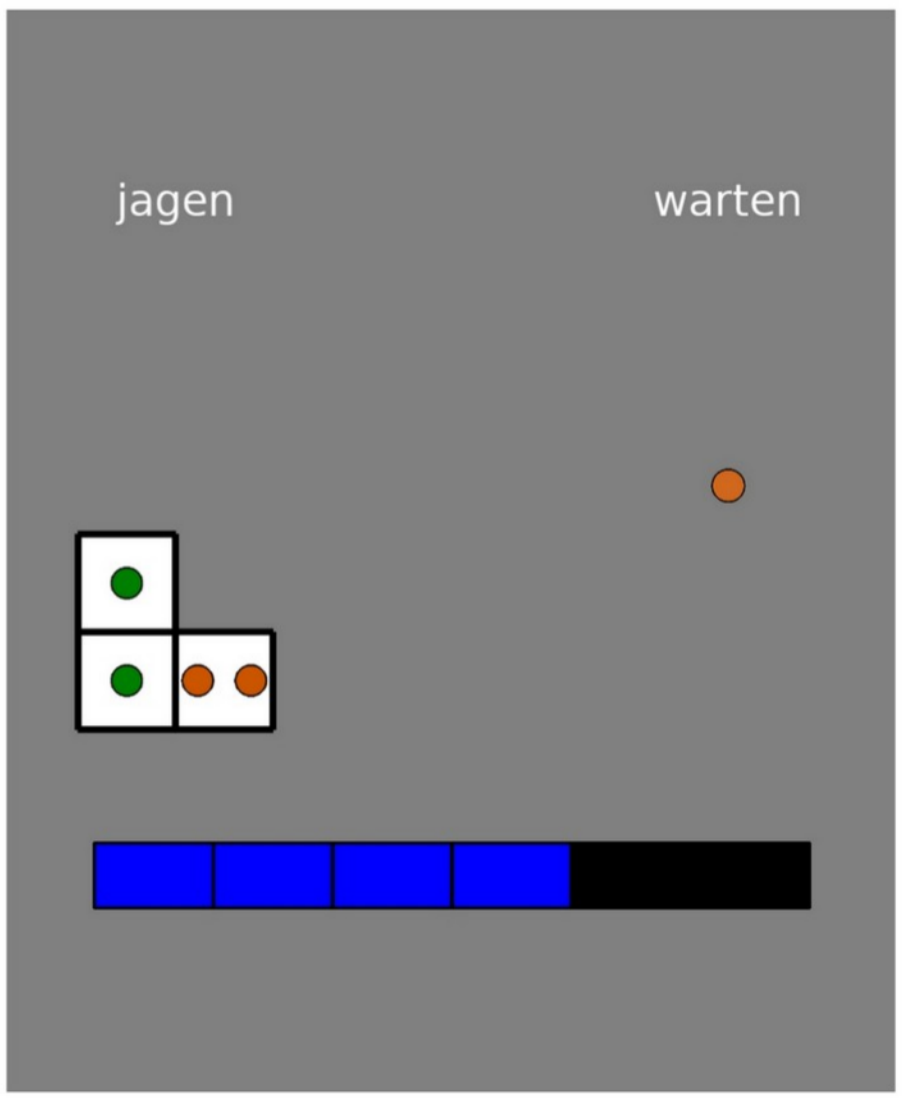

Example 2, Day 1: Choices

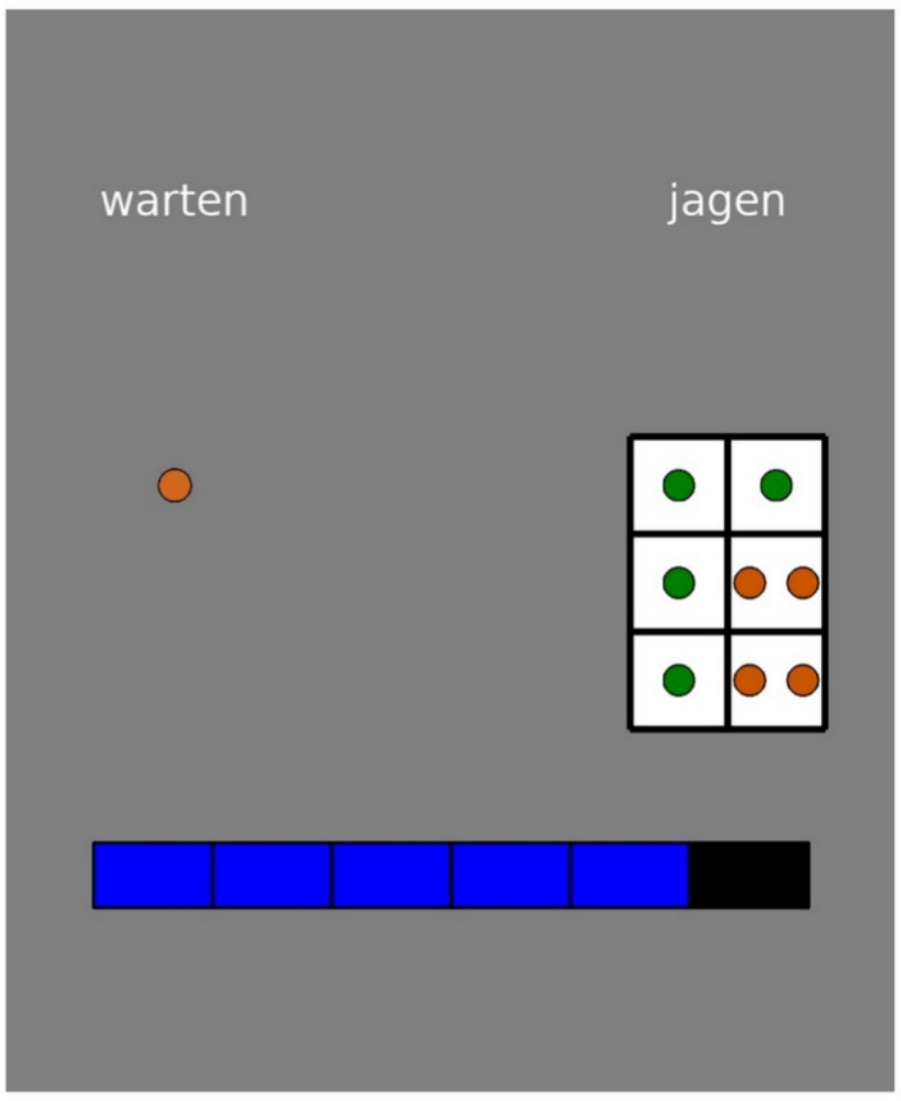

- If you decide to go foraging/hunting, you will land randomly on one of the fields, whereby the probability is the same for each field. For example, if an environment consists of 4 fields, you have a 25% chance of landing on one of the fields.
- If you land on a field with food during the hunt/search, you have been successful. In this case, the corresponding field lights up light green. You will then receive life points. The amount of loot for a successful hunt/search is +1 or +2 life points. This is shown with either 1 or 2 green dots.
- If you land in a square with two red dots, your hunt is unsuccessful. In this case, the corresponding field lights up pink and you lose -2 life points.

Outcome hunt/search, example 1: success

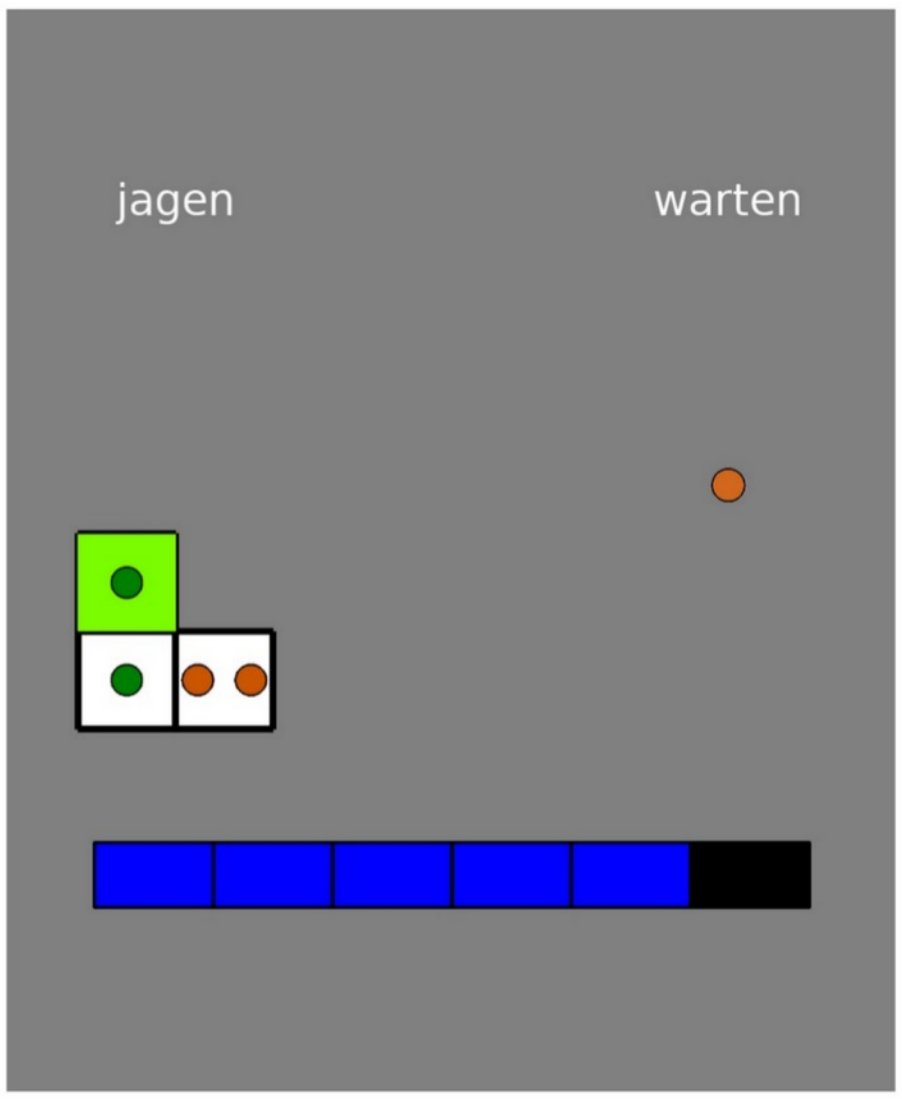

Outcome hunt/search, example 2: fail

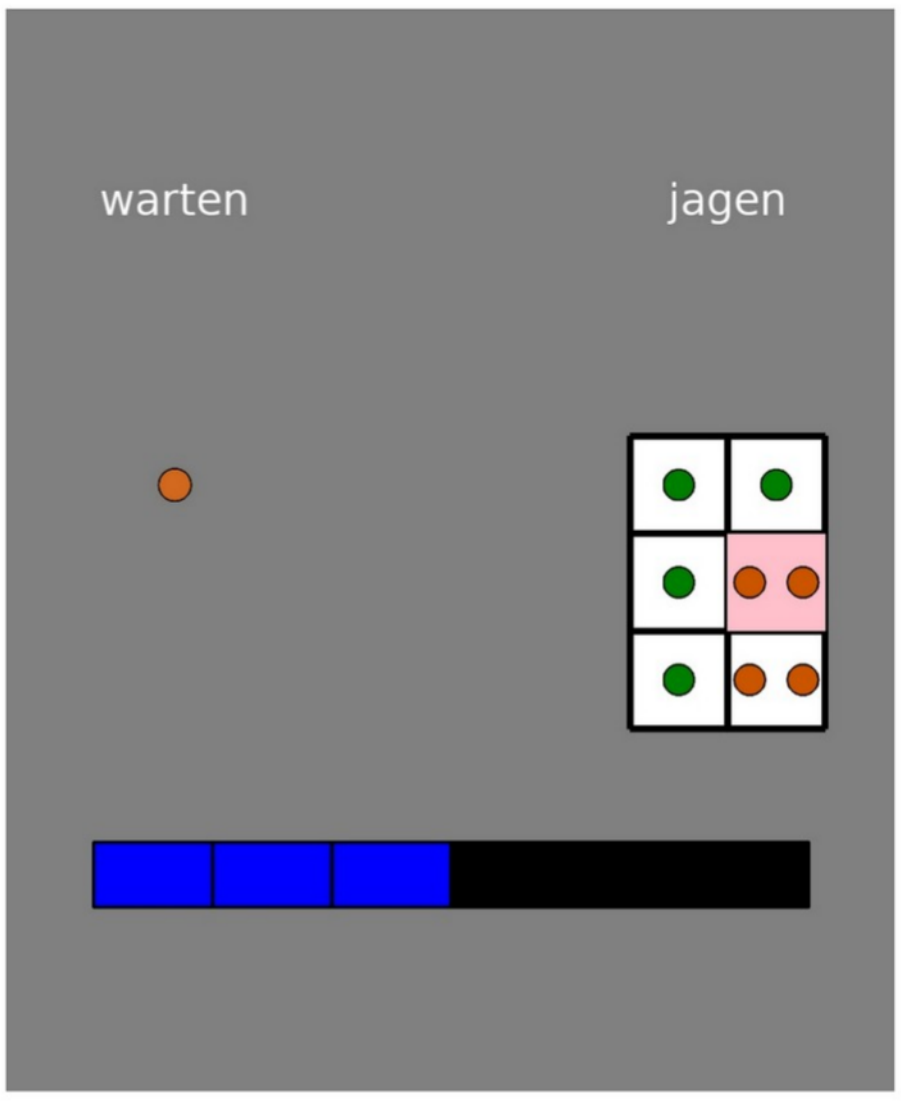

- If you do not hunt/search or wait, you will certainly lose -1 life point. A single red dot indicates this option. This decision is indicated by a chiffon yellow field.

ATTENTION: If you take more than 3 seconds to make your decision, you have hesitated too long and the wait option is automatically selected. You will lose -1 life point. If you deliberately decide to wait, PLEASE use the arrow key and do not simply let the time run down, otherwise your data for the affected runs cannot be recorded.

Example of the display after the 'wait' decision: You receive -1 life point

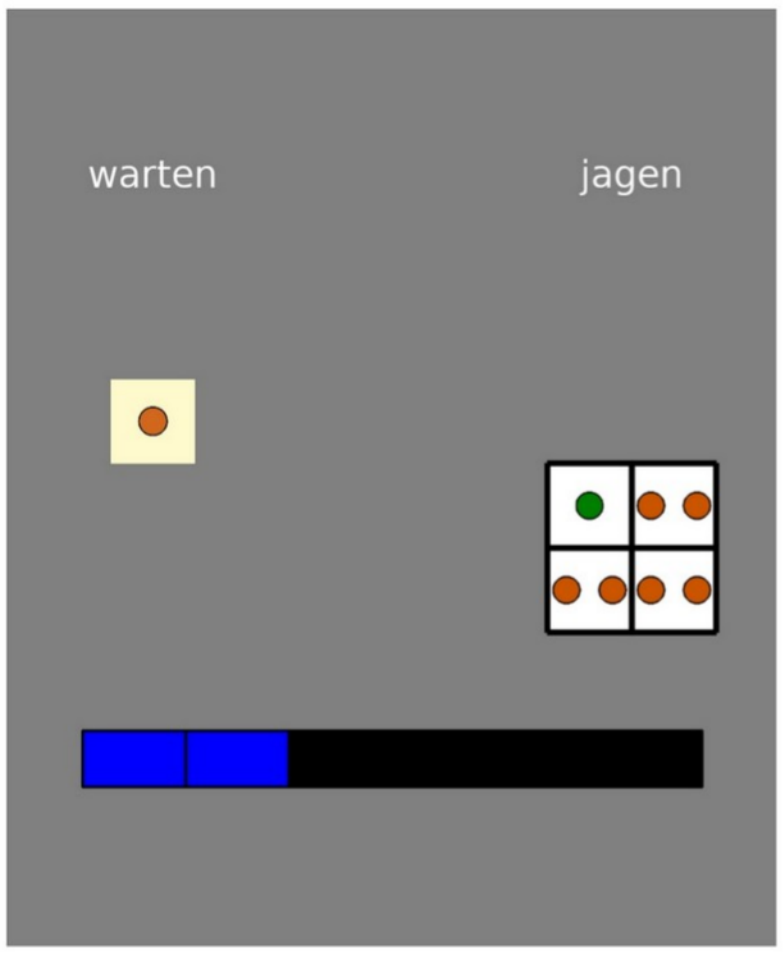

- Either 0, 1 or 2 predators can move in the same environment as you. This is shown with the corresponding number of randomly flashing red edges of the fields in a weather condition.
- Each predator has the same chance as you to land on one of the fields of a weather condition. If you happen to land in the same square as a predator, the corresponding square lights up in magenta and you lose -3 life points.

Example of forest with predator:

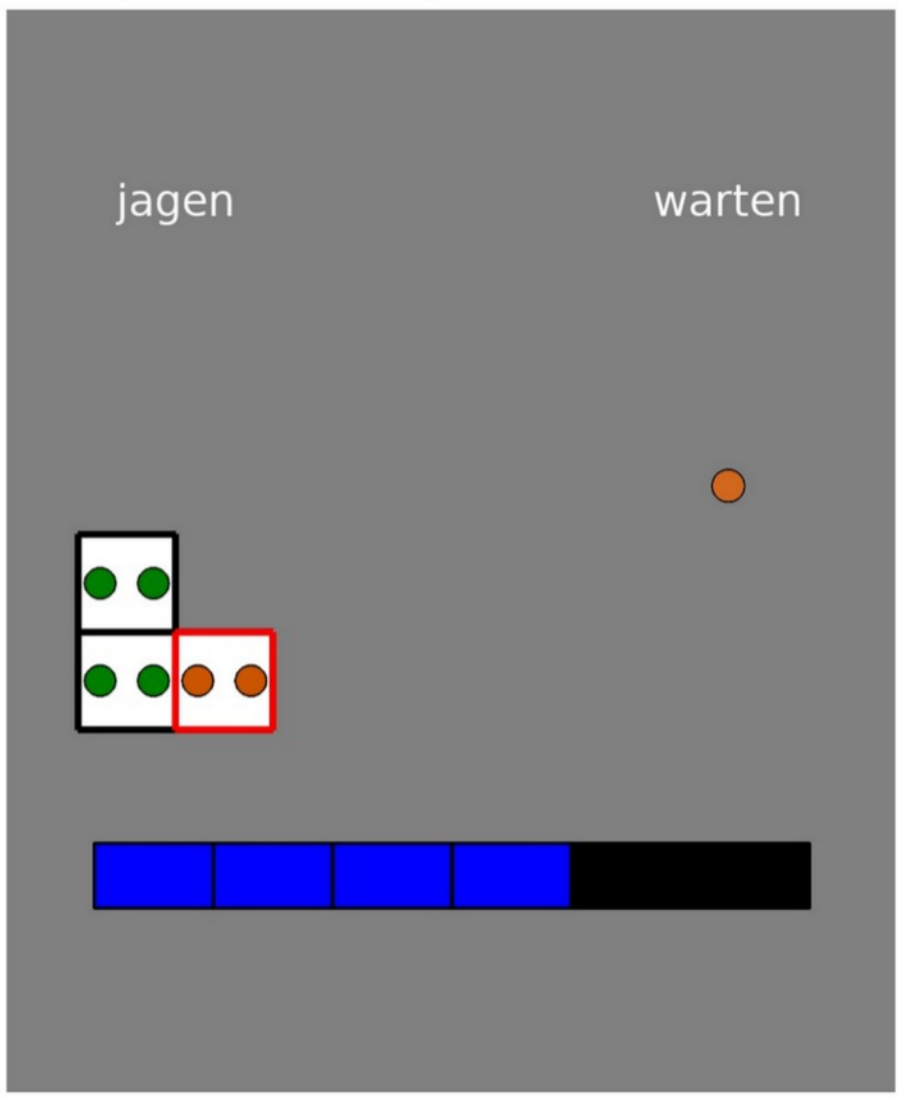

Encountering predator during hunt:

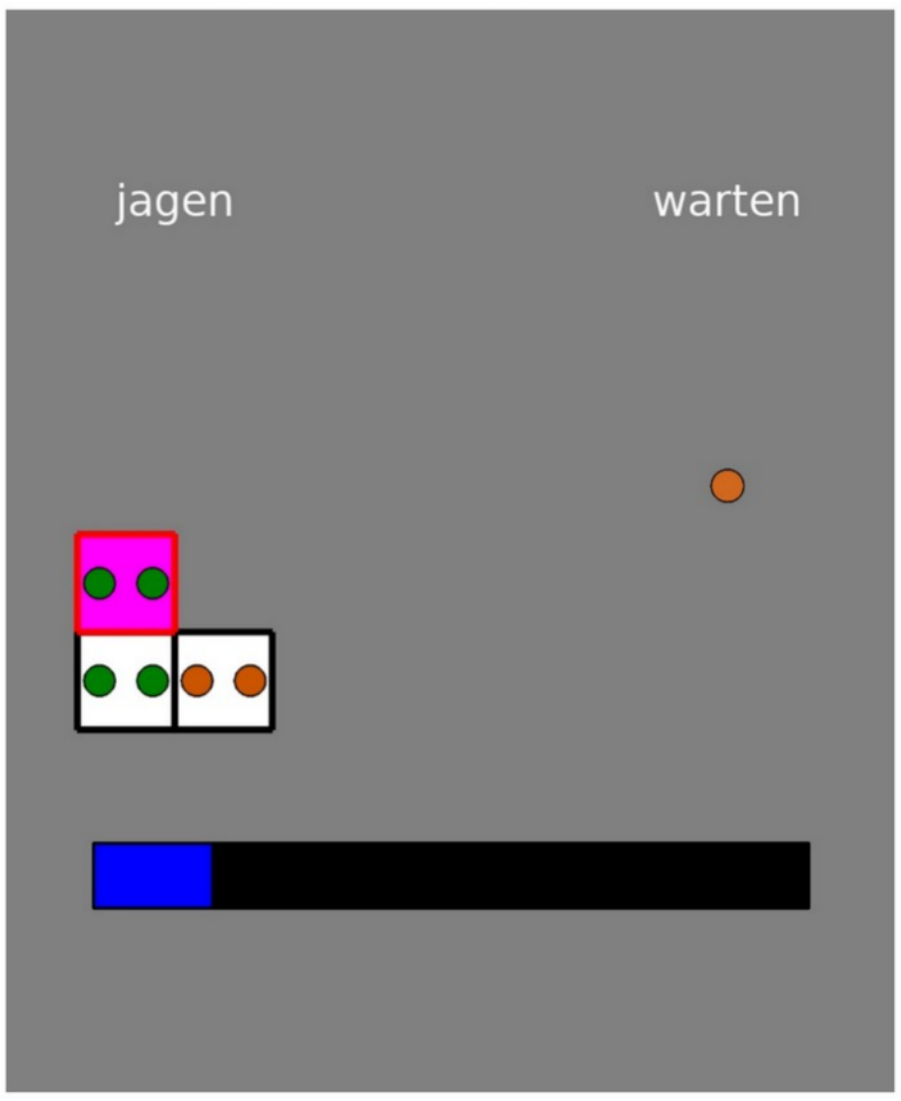

- After the result of a decision has been presented, a new day with the same or a different weather condition will appear, and you will have to choose the options wait or hunt/search again. If you do not starve to death beforehand, this will happen eight times in a row before you are automatically taken to a new forest with two new weather conditions.
- If your life points drop to 0 before the end of a round, you die and immediately enter a new forest with two new weather conditions.

Additional information:

Keys: Select the left option with the left arrow key and the right option with the right arrow key.

Extra payoff: You play several blocks. Three rounds are randomly selected from each block. For each of these selected rounds in which you survive to the end, you receive an additional **50 cents**.

You will first complete a short training session so that you can see how the game works.

If you have any questions, please contact the principal investigator.
