# Supplementary material for: Context-dependent feature modulation shapes human decision policies in approach–avoidance conflicts
Source: PLoS Comput Biol. 2026 Sep 30;22(9):e1014793. doi: 10.1371/journal.pcbi.1014793 (PMC13652344; doi:10.1371/journal.pcbi.1014793)
Supplement: Supplementary file 2 [file pcbi.1014793.s002.pdf]

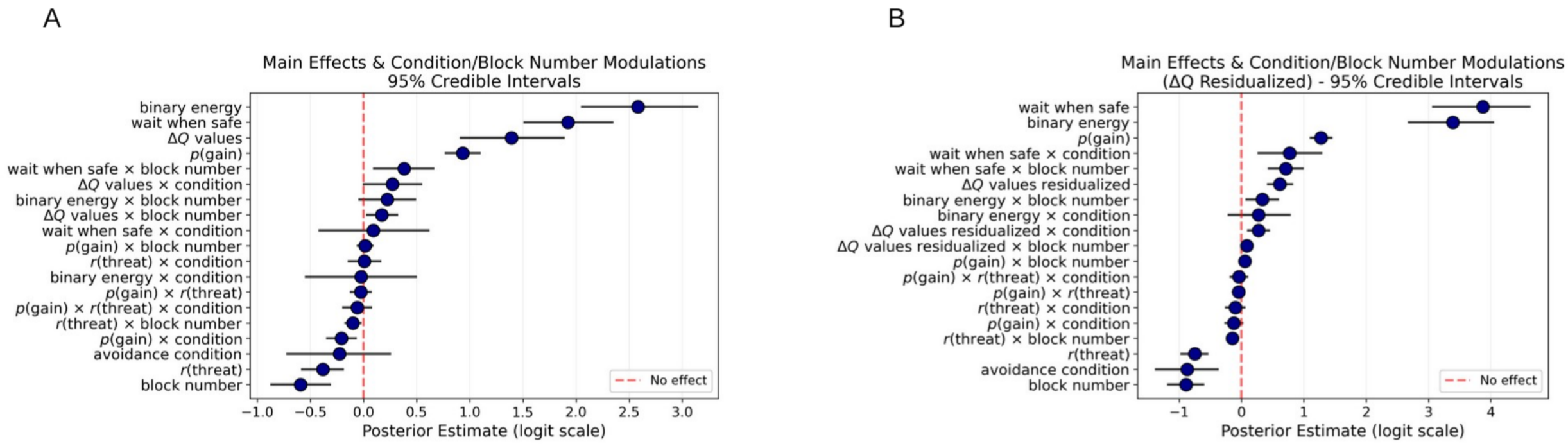

S1 Fig. Full hierarchical moderation model: main effects and interaction terms.

Posterior mean estimates (points) and 95% credible intervals (horizontal lines) are shown for all main effects and interaction terms from the hierarchical logistic regression model. Estimates are reported on the logit scale; positive values indicate an increased probability of choosing the foraging option, whereas negative values indicate a decreased probability. The vertical dashed line denotes the null effect (0). ***(A)*** *Model including raw ΔQ.* ***(B)*** *Model including ΔQ residualized with respect to the variables of the multi-feature model.*

Main effects reflect baseline contributions of decision variables, including reward probability (p(gain)), threat probability (r(threat)), model-derived optimal values (ΔQ), and state-dependent features (binary energy, wait when safe). Interaction terms capture how these contributions are modulated by experimental condition (approach vs. avoidance) and block number. Across both specifications, results indicate selective feature reweighting rather than global shifts (no condition main effect). In avoidance contexts, the influence of p(gain) is reduced, whereas alignment with ΔQ increases, while sensitivity to r(threat) remains comparatively stable. Block-related interactions indicate gradual changes in feature weighting over time, characterized by increasing alignment with ΔQ and boundary states (binary energy state and wait when safe).

Crucially, the residualized model in ***(B)*** *confirms that the condition-dependent increase in ΔQ persists beyond variance shared with other predictors, indicating a unique contribution of integrated value representations. Higher-order interactions, including p × r and p × r × condition, were not credibly different from zero in either model, suggesting that explicit feature interactions do not explain behavior beyond integrated value signals captured by ΔQ. Continuous predictors were standardized prior to model fitting. Condition was coded as 0 = approach and 1 = avoidance.*
