# Supplementary material for: Context-dependent feature modulation shapes human decision policies in approach–avoidance conflicts
Source: PLoS Comput Biol. 2026 Sep 30;22(9):e1014793. doi: 10.1371/journal.pcbi.1014793 (PMC13652344; doi:10.1371/journal.pcbi.1014793)
Supplement: Supplementary file 3 [file pcbi.1014793.s003.pdf]

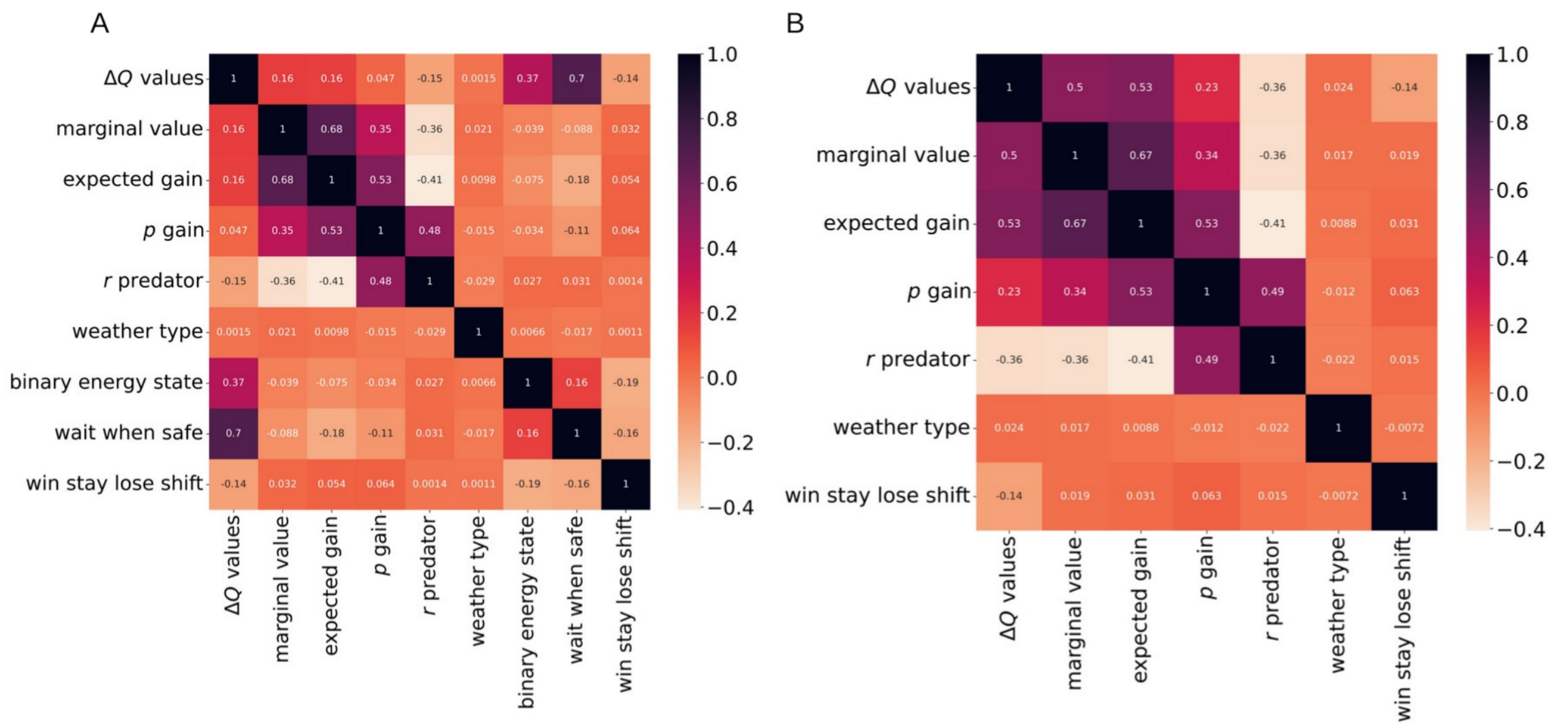

S2 Fig. Correlation structure of model features across all trials and trade-off trials.

Pairwise Pearson correlations between optimal policy values (ΔQ) and candidate decision variables. Color intensity indicates correlation strength (−1 to 1). ***(A)*** *Correlations across all trials. ΔQ values show relatively weak associations with individual environmental features (e.g., p(gain): r = 0.05; r(predator): r = −0.15), but stronger correlations with state-dependent variables, particularly wait when safe (r = 0.70) and binary energy state (r = 0.37). Several heuristic and value-based predictors are more strongly intercorrelated (e.g., marginal value and expected gain: r = 0.68), reflecting shared structure among simpler decision variables.* ***(B)*** *Correlations restricted to trade-off trials (|ΔQ| near zero). In these difficult decision states, associations between ΔQ and value-based predictors increase (e.g., expected gain: r = 0.53; marginal value: r = 0.50), while correlations with state-dependent features are substantially reduced or absent.*

The multi-feature policy was not included, as it is a composite function of the predictors and therefore shares variance with them by construction. Correlations between such composite outputs and their constituent features are not informative for assessing multicollinearity among predictors. Accordingly, this analysis is intended as a descriptive check for dependencies among candidate features and does not replace formal model comparison or model recovery procedures.

Overall, the pattern indicates that correlations observed across all trials are partly driven by extreme (easy) states, whereas in trade-off trials, predictors align more strongly with value-based representations, consistent with increased alignment with integrated value signals in difficult decisions.
