# Supplementary material for: Context-dependent feature modulation shapes human decision policies in approach–avoidance conflicts
Source: PLoS Comput Biol. 2026 Sep 30;22(9):e1014793. doi: 10.1371/journal.pcbi.1014793 (PMC13652344; doi:10.1371/journal.pcbi.1014793)
Supplement: Supplementary file 4 [file pcbi.1014793.s004.pdf]

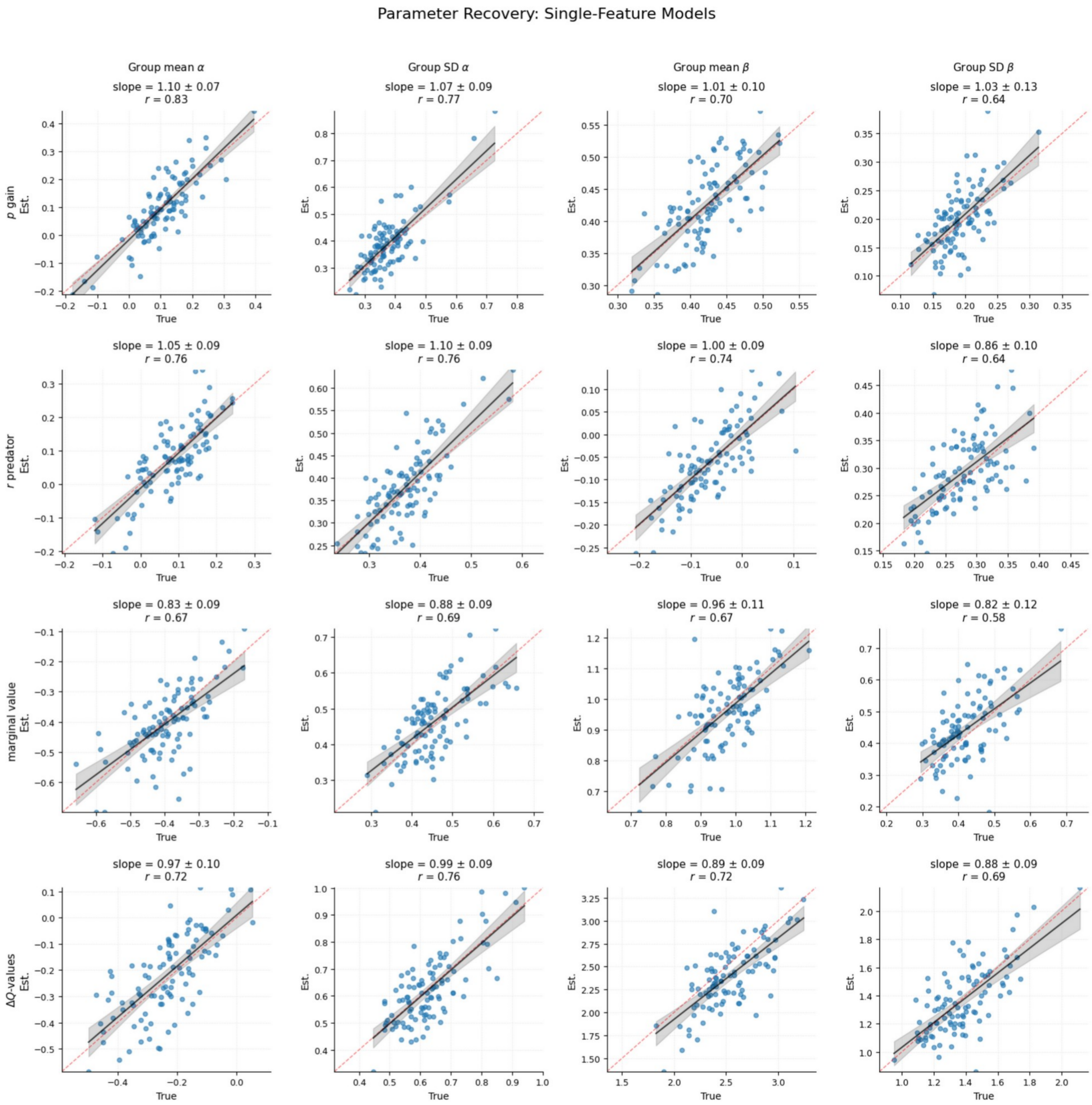


***S3 Fig. Parameter recovery results for the hierarchical Bayesian single-feature policy models.*** *For each candidate model, 100 synthetic datasets were generated from posterior parameter estimates of the empirically fitted models and subsequently refit using the corresponding hierarchical model specification. For clarity and readability, the figure displays representative single-feature models spanning the principal classes of candidate policies; recovery performance for the remaining models showed comparable overall patterns. Recovery analyses used conditional simulation procedures in which choices were regenerated from the fitted models while preserving the original trial structure, task states, and stimulus configurations of the empirical dataset. Scatterplots depict the relationship between true generating parameters and recovered posterior mean estimates across simulations. Columns show recovery of the group mean intercept parameters (mean α), group-level intercept variability (SD α), group mean slope parameters / feature weights (mean β), and group-level slope variability parameters (SD β). The dashed red line indicates the identity line corresponding to perfect recovery, while the solid black line shows the fitted regression line with 95% confidence intervals. Recovery performance was quantified using regression slopes and Pearson correlations between true and recovered parameter values. Across models, parameter recoveries demonstrated generally good calibration and moderate-to-strong correlations, indicating reliable recovery of both central tendency and inter-individual variability parameters. Recovery analyses used the same hierarchical Bayesian logistic regression framework as the empirical analyses, including non-centered parameterization, weakly informative priors, and partial pooling across participants. Recovery fits were estimated using 4 Markov chains with 500 tuning and 500 posterior sampling iterations per chain (target_accept = 0.95). Convergence diagnostics produced frequent $\hat{R}$ and effective sample size warnings for datasets generated by very simple single-feature policies, particularly when these datasets were fit using more flexible hierarchical models. This likely reflects weak parameter identifiability and quasi-separable synthetic choice patterns arising from highly deterministic low-dimensional policies rather than failures of overall model recovery. Despite these warnings, the recovery analyses showed robust model discriminability and reliable recovery of the principal group-level parameters.*
