# Supplementary material for: Context-dependent feature modulation shapes human decision policies in approach–avoidance conflicts
Source: PLoS Comput Biol. 2026 Sep 30;22(9):e1014793. doi: 10.1371/journal.pcbi.1014793 (PMC13652344; doi:10.1371/journal.pcbi.1014793)
Supplement: Supplementary file 5 [file pcbi.1014793.s005.pdf]

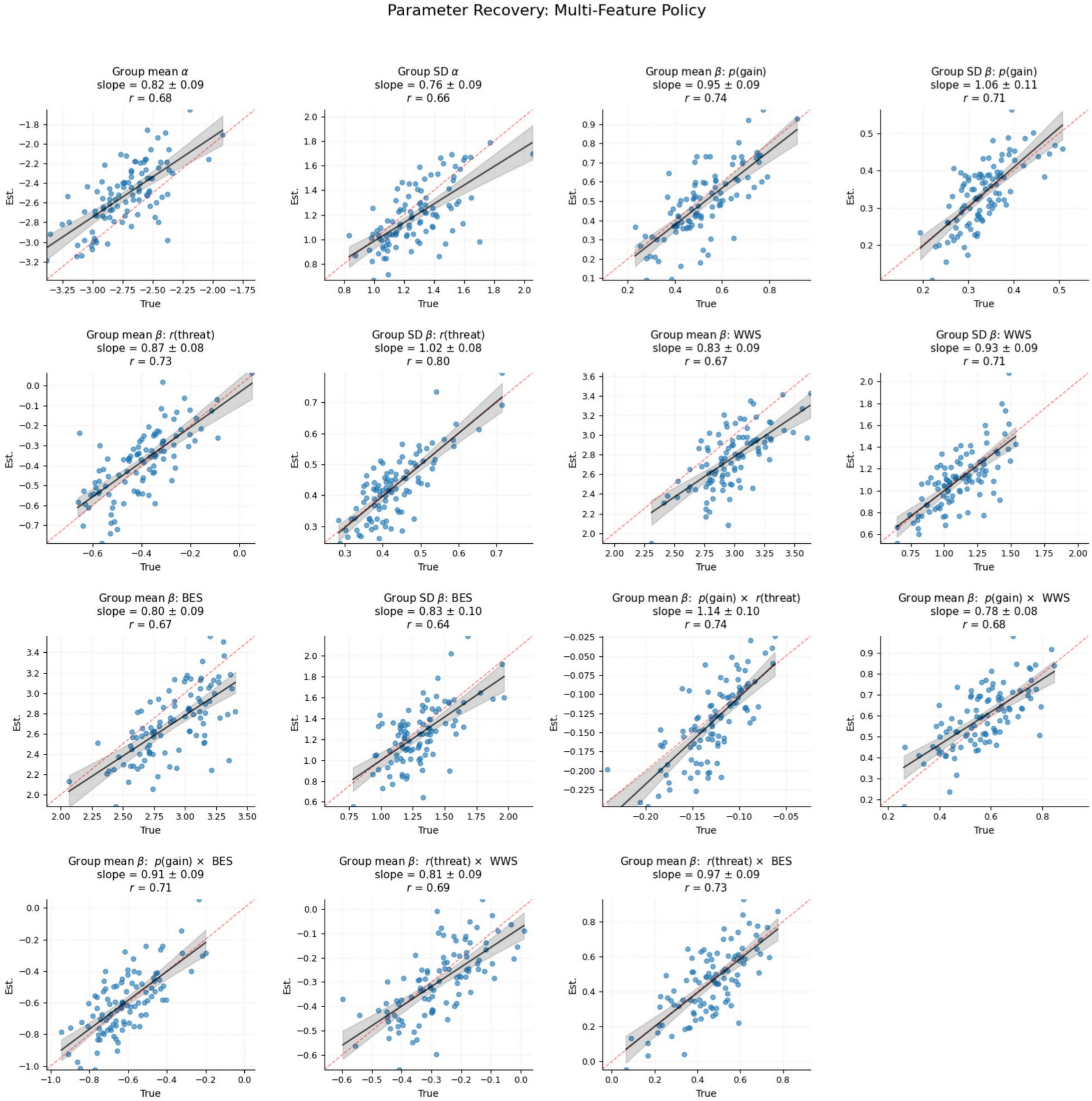

S4 Fig. Parameter recovery results for the hierarchical Bayesian multi-feature policy model. *For each simulation, synthetic datasets were generated from posterior parameter estimates of the empirically fitted model and subsequently refit using the same hierarchical model specification. Recovery analyses used conditional simulation procedures in which choices were regenerated from the fitted policy while preserving the original trial structure, task states, and stimulus configurations of the empirical dataset. Scatterplots depict the relationship between true generating parameters and recovered posterior mean estimates across simulations. Panels show recovery of the group mean intercept parameters (mean α), group-level intercept variability (SD α), group mean slope parameters / feature weights (mean β), and corresponding group-level variability parameters (SD β) for the main effects and interaction terms included in the multi-feature policy model. The interaction between "wait when safe" (WWS) and "binary energy state" (BES) was excluded from the model because these predictors represented mutually exclusive boundary-state conditions by task design, and inclusion of the interaction introduced structurally redundant parameterization that reduced parameter recoverability without improving model expressiveness. Interaction coefficients are denoted using × between interacting predictors. The dashed red line indicates the identity line corresponding to perfect recovery, while the solid black line shows the fitted regression line with 95% confidence intervals. Recovery performance was quantified using linear regression slopes and Pearson correlations between true generating and recovered parameter estimates. Across parameters, recoveries demonstrated generally good calibration and moderate-to-strong correlations, indicating reliable identifiability of the principal feature weights and interaction effects of the multi-feature policy. Recovery analyses used the same hierarchical Bayesian logistic regression framework as the empirical analyses, including non-centered parameterization, weakly informative priors, partial pooling across participants, and all interaction terms specified in the fitted model. Recovery fits were estimated using 4 Markov chains with 500 tuning and 500 posterior sampling iterations per chain (target_accept = 0.95). As in the single-feature recovery analyses, convergence diagnostics occasionally produced elevated $\hat{R}$ values and reduced effective sample sizes, particularly for highly deterministic simulated datasets and interaction parameters. However, these issues did not materially affect overall parameter recoverability or model discriminability.*
