# Supplementary material for: Context-dependent feature modulation shapes human decision policies in approach–avoidance conflicts
Source: PLoS Comput Biol. 2026 Sep 30;22(9):e1014793. doi: 10.1371/journal.pcbi.1014793 (PMC13652344; doi:10.1371/journal.pcbi.1014793)
Supplement: Supplementary file 6 [file pcbi.1014793.s006.pdf]

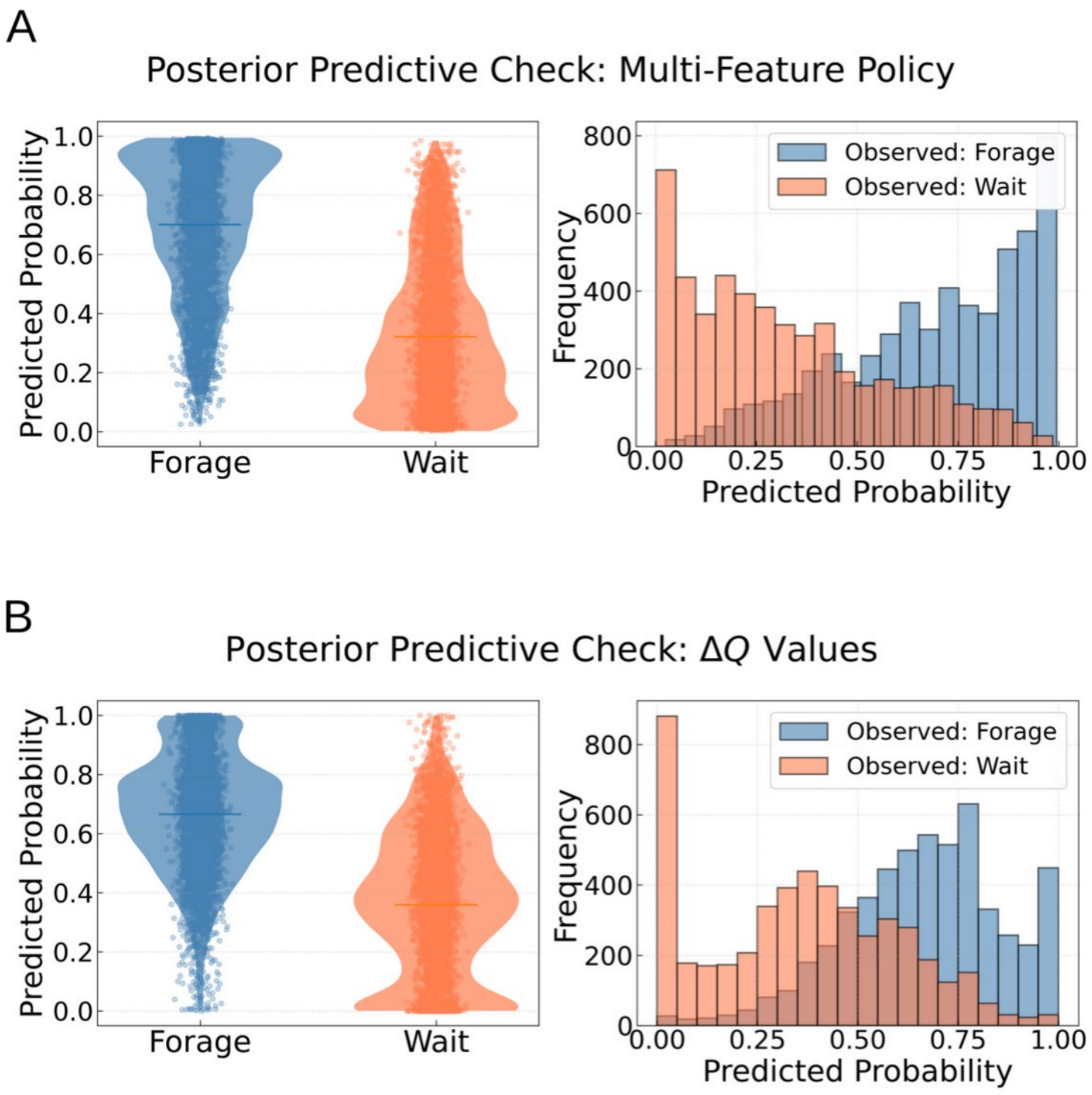

S5 Fig. Posterior predictive checks for the primary inferential models.

Posterior predictive distributions for ***(A)*** *the multi-feature policy model and* ***(B)*** *the ΔQ value model. Left panels show predicted choice probabilities for observed forage and wait decisions across all trials. Right panels show the corresponding distributions of predicted probabilities conditional on the observed behavioral choice. Both models reproduced the overall structure of participant behavior, with observed forage decisions generally associated with higher predicted foraging probabilities and observed wait decisions associated with lower predicted foraging probabilities. The multi-feature policy model showed stronger probabilistic separation between observed choice categories, consistent with its superior predictive performance in PSIS-LOO model comparison.*
