# Supplementary material for: Context-dependent feature modulation shapes human decision policies in approach–avoidance conflicts
Source: PLoS Comput Biol. 2026 Sep 30;22(9):e1014793. doi: 10.1371/journal.pcbi.1014793 (PMC13652344; doi:10.1371/journal.pcbi.1014793)
Supplement: Supplementary file 7 [file pcbi.1014793.s007.pdf]

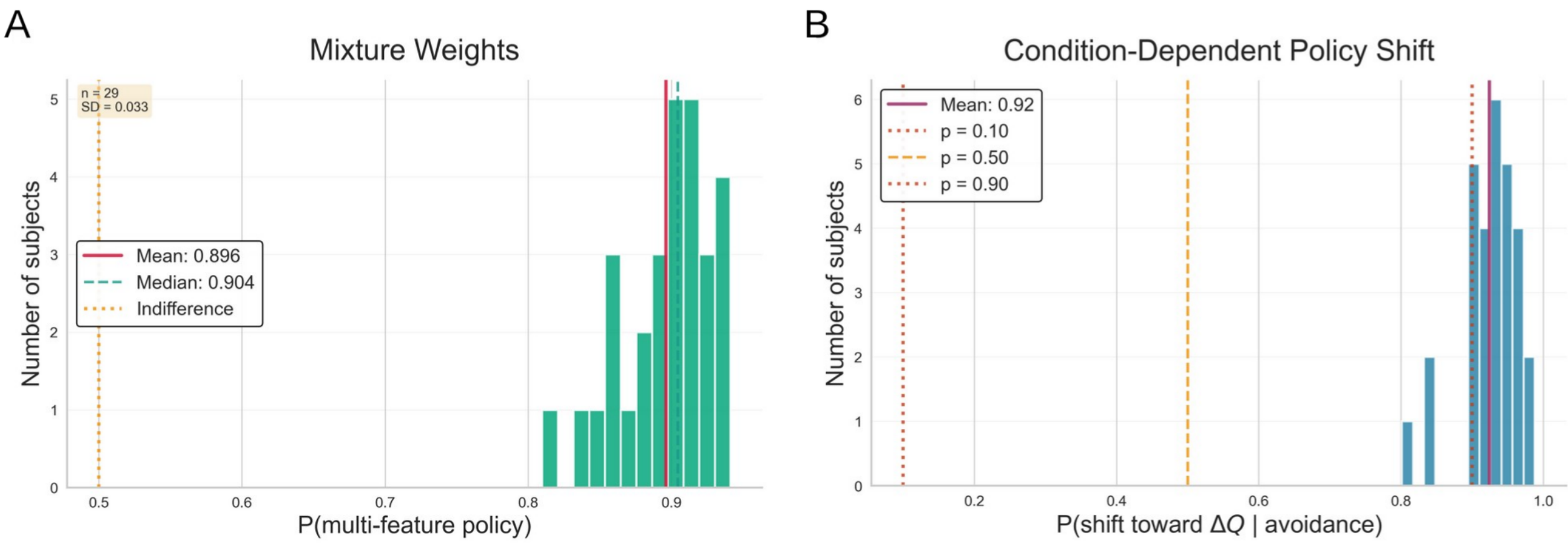


***S6 Fig. Exploratory hierarchical policy-arbitration analysis examining continuous alignment with the multi-feature (MF) policy versus integrated ΔQ value representations across individuals and conditions.*** *A hierarchical Bayesian continuous mixture model was used to estimate trial-wise arbitration weights ($\pi_t$) reflecting the relative contribution of the MF versus ΔQ-based policy representations to observed choices. At the trial level, action probabilities were modeled as a probabilistic mixture of the two policy predictions:*

$$P(a_t) = \pi_t P_{MF}(a_t) + (1- \pi_t)P_{\Delta Q}(a_t),$$

*where $P_{MF}(a_t)$ and $P_{\Delta Q}(a_t)$ denote the trial-wise action probabilities predicted by the MF and ΔQ-based models, respectively, and $\pi_t$ represents the arbitration weight favoring the MF policy. Importantly, the present analysis was designed to examine policy-level arbitration rather than feature-level modulation. Accordingly, both policy representations were first estimated independently using the hierarchical models from the primary analyses, and the resulting posterior action probabilities were then treated as fixed policy predictions within the arbitration model. This approach allowed arbitration tendencies to be examined while treating the independently estimated policy predictions as fixed inputs to the arbitration model.*

*The ΔQ-based policy representation was operationalized using posterior predictions derived from the fitted Markov Decision Process (MDP) model based on integrated state–action value differences (ΔQ). The MF policy representation was operationalized using posterior predictions derived from the fitted hierarchical multi-feature model integrating reward probability (p(gain)), threat probability (r(threat)), and the boundary-condition features binary energy state (BES) and wait when safe (WWS). Arbitration weights were estimated hierarchically across participants and task conditions (approach vs. avoidance) while additionally controlling for block number and trial-wise policy similarity. Trial-wise policy similarity was quantified using the absolute difference between the MF and ΔQ-based policy predictions ($\Delta_t = |P_{\Delta Q} - P_{MF}|$), thereby accounting for trial-wise variation in overlap between the competing policy accounts. Importantly, policy-similarity distributions were highly comparable across conditions, indicating that the observed condition-dependent arbitration effects were unlikely to be explained solely by differences in overlap between the candidate policy representations across approach and avoidance environments.*

***(A)*** *Distribution of baseline arbitration weights across participants. Most participants showed greater overall alignment with the MF policy representation, consistent with the primary model-comparison analyses reported in the main text. However, arbitration weights formed a continuous distribution without evidence for sharply separable strategy classes, suggesting graded inter-individual variability in relative policy alignment.*

***(B)*** *Posterior probabilities of condition-dependent shifts toward increased ΔQ-based policy alignment in avoidance contexts. Most participants showed posterior probabilities substantially above 0.5, indicating a consistent directional tendency toward greater alignment with integrated-value representations under avoidance conditions despite substantial overlap between the MF and ΔQ-based policy predictions.*
