# Supplementary material for: Context-dependent feature modulation shapes human decision policies in approach–avoidance conflicts
Source: PLoS Comput Biol. 2026 Sep 30;22(9):e1014793. doi: 10.1371/journal.pcbi.1014793 (PMC13652344; doi:10.1371/journal.pcbi.1014793)
Supplement: Supplementary file 8 [file pcbi.1014793.s008.pdf]

Dear Editor and Reviewers,

We sincerely thank the editor and both reviewers for their careful, thoughtful, and constructive evaluation of our manuscript. Their comments substantially improved the conceptual clarity, methodological rigor, and interpretability of the work.

In response to the reviews, we have revised the manuscript extensively. A central theme of the revision was to more clearly distinguish normative optimality from psychological processes and to evaluate whether the observed behavioral effects are consistent with context-dependent changes in "feature weighting" rather than a global shift toward a qualitatively different decision strategy. To address the reviewers' concerns, we refined the theoretical framing, expanded robustness and control analyses, strengthened the computational modeling framework, and substantially revised the discussion of alternative explanations and limitations.

The main changes include:

Methodological Rigor & Robustness

- Transition from single-subject estimation to a hierarchical Bayesian modeling framework.
- Replacement of the original BIC/Bayes-factor-based model comparison with PSIS-LOO cross-validation to evaluate out-of-sample predictive performance.
- Addition of convergence diagnostics, posterior predictive checks, model recovery analyses, and parameter recovery analyses.
- Inclusion of multiple control analyses addressing alternative explanations, including task difficulty, response times, learning effects, policy overlap, and environmental differences between conditions.

Conceptual Framing & Terminology

- Clear distinction between MDP-derived ΔQ-values as a normative benchmark and the psychological processes underlying behavior.
- Reframing of the main interpretation from a shift toward a more "optimal" or "sophisticated" strategy to context-dependent modulation and integration of decision-relevant features.
- Renaming of the "multi-heuristic" model as the "multi-feature" model to better reflect its interpretation.
- Revision of the title to: *Context-dependent feature modulation shapes human decision policies in approach–avoidance conflicts.*

Narrative Clarity

- Substantial restructuring of the Introduction to better integrate reinforcement-learning and behavioral-ecology perspectives.
- Expanded discussion of limitations, including sample size, symbolic threat manipulation, learning and fatigue effects, and the interpretational limits of normative policy benchmarks.
- Addition of exploratory analyses examining individual differences in alignment between feature-based and integrated value-based policy representations.

Overall, the revised manuscript preserves the central empirical findings while providing a more precise mechanistic interpretation and a substantially expanded set of robustness analyses. We

believe these revisions have substantially strengthened the manuscript and have addressed the major concerns raised by both reviewers.

Below, we respond point-by-point to all reviewer comments. Reviewer comments are reproduced in italics, followed by our responses.

Sincerely,

Sergej A. E. Golowin
Niall W. Duncan
Christoph W. Korn

Reviewer #1

"I would like to thank the editor for the opportunity to review this manuscript. The study tackles a timely and important question about how humans dynamically adjust their decision-making strategies in threatening versus neutral environments. By combining an ecologically motivated approach–avoidance paradigm with formal model comparison, this work addresses a significant gap in understanding how environmental threat modulates policy sophistication. While I find the approach valid and original, I have some minor reservations concerning actual sample size and general robustness checks."

Response:
We sincerely thank Reviewer #1 for the positive assessment and thoughtful evaluation of our manuscript. We are pleased that the reviewer finds the work timely and relevant and views the paradigm and modeling approach as a valuable contribution to understanding context-dependent adaptation in sequential decision-making. We greatly appreciate the constructive feedback, which helped strengthen the manuscript. Detailed responses to the individual comments are provided below.

Introduction

Comment 1

"The introduction is conceptually strong and well written. It clearly situates the study within the context of model-based versus model-free decision-making and frames the question through an ecological and evolutionary lens. The authors do an excellent job connecting dual-process frameworks with behavioral ecology concepts such as bounded rationality and meta-decision making."

Response:
We sincerely thank the reviewer for this positive assessment. We are pleased that the reviewer found the conceptual framing and integration of decision-making, behavioral ecology, and bounded-rationality perspectives to be a strength of the manuscript. In the revised version, we further refined this theoretical framework by clarifying the distinction between normative optimality and psychological processes and by more clearly positioning the MDP-derived ΔQ-values (the optimal state–action value difference, $\Delta Q = Q_{forage} - Q_{wait}$) as a normative benchmark for evaluating adaptive behavior. We also strengthened the discussion of how context-dependent adaptation may emerge through changes in the weighting and integration of decision-relevant features within a shared

decision architecture. For the sake of brevity, the relevant parts of the Introduction are copied below in response to other comments.

Comment 2

"Still, I must point out that I personally find this introduction generally dense, and somewhat dissagregated regarding the other topics it touches upon. The flow between the reinforcement learning literature (Daw et al.) and ecological foraging literature (Kolling, Mobbs, etc.) could be more cohesive, since at times these threads feel like parallel lines rather than something that has been theoretically integrated."

Response:

We agree with the reviewer that the original Introduction did not fully integrate the reinforcement-learning and behavioral-ecology perspectives. In the revised manuscript, we substantially restructured and streamlined the Introduction to strengthen the conceptual links between these literatures. Rather than presenting them as parallel frameworks, we now emphasize their shared focus on adaptive decision-making in sequential environments and explicitly connect sequential foraging, approach-avoidance conflict, and reinforcement learning concepts through the notion of decision policies.

To make this integration more explicit, we now frame sequential accept-reject decisions as a common computational problem that links foraging and approach-avoidance behavior. We further introduce decision policies as a shared framework for understanding how organisms adapt behavior under uncertainty, while clarifying the distinction between integrated value representations and feature-based decision policies. For example, the revised Introduction now states:

"Integrating sequential decision-making with AAC is particularly valuable because adaptive behavior requires both anticipating future consequences and balancing competing gains and risks. Sequential choice paradigms provide a powerful framework for studying these processes because they allow behavior to be analyzed in terms of decision policies, that is, rules mapping states onto actions over time. Such policies may depend either on integrated value computations or on simpler decision-relevant features derived from observable environmental cues, such as reward probability or threat risk. In this sense, individual features can guide behavior through simple feature-based decision rules (...). Policies based on integrated values, on the other hand, can be formalized within Markov Decision Processes (MDPs), which specify value-maximizing behavior across temporally extended choice sequences (...). (...) Distinguishing between behavior that arises from integrated value representations and behavior that emerges through adaptive weighting of decision-relevant features is important because superficially similar adaptive behavior may arise from fundamentally different computational mechanisms, with distinct implications for theories of decision-making in psychology and economics." (Lines 13-38)

These revisions provide a more cohesive theoretical foundation linking reinforcement learning, behavioral ecology, and approach–avoidance decision-making.

Comment 3

"While the hypothesis is clearly stated (if I follow correctly, that one should anticipate 'greater policy sophistication' under threat), the whole section might benefit from an explicit operational definition of 'sophistication' earlier on, i.e. whether this refers to model-based computation, policy flexibility, or response adaptation."

Response:
We thank the reviewer for highlighting this important conceptual issue. We agree that the original use of the term "policy sophistication" was ambiguous and risked conflating normative optimality with process-level claims about model-based computation or qualitatively different decision strategies. In the revised manuscript, we therefore removed this terminology and replaced it with a more explicit distinction between alignment with the optimal policy and the computational mechanisms that generate behavior.

Specifically, we now clarify that behavior may seem near-optimal, while the computational mechanism underneath may be based on simpler, more direct environmental feature evaluation. This point is specifically addressed here:

"Importantly, behavior need not rely on explicit value maximization to appear near-optimal. Similar adaptive behavior may also emerge through selective weighting of informative decision features, that is, through changes in how strongly decision-relevant features influence choice, allowing adaptive policies to be approximated without explicit computation of exhaustive value representations. Such lower-dimensional policy representations may provide a computationally scalable and flexible basis for adaptation *(...)." (Lines 30-35)*

Accordingly, we revised both the theoretical synthesis and reformulated the hypotheses in this nomenclature to avoid framing contextual adaptation as a shift toward a more "sophisticated" or explicitly model-based strategy. The revised manuscript makes clear that we focus on the computational mechanism allowing adaptive behavior, for which we adopted approach-avoidance contexts in our experiment:

"Within AAC, contextual variation emerges from how reward and threat jointly determine the long-term value of available actions. (...) Recent accounts suggest that this flexibility may arise not through switches between entirely distinct decision strategies, but through context-dependent changes in reliance on specific decision features (...). Under this view, approach and avoidance contexts may shape behavior by selectively modulating the relative influence of specific decision features, such as reward probability or threat risk, while preserving a shared underlying computational process." (Lines 40-52)

"(…) (1) whether approach and avoidance contexts differ in the extent to which behavior aligns with optimal policies, and (2) whether such differences can be explained by context-dependent modulation of decision-relevant features." (Lines 66-69)

Methods

Comment 1

"The methodological design is thoughtful and rigorous. The 'hunter-gatherer' paradigm is a creative and ecologically valid extension of previous work by Korn & Bach. The MDP formalization is well justified, and the implementation of 'approach' and 'avoidance' forests is elegant in manipulating threat without confounding motivation and reward structure."

Response:
We thank the reviewer for this positive evaluation of the task design and modeling framework.

In the revised manuscript, we further clarified both the rationale underlying the threat manipulation and the relationship between the task design and the computational modeling framework.

Specifically, we now emphasize that threat-related outcomes were implemented symbolically through probabilistic predator encounters rather than direct aversive stimulation, while still imposing substantial survival costs within the task structure. We also expanded the description of the modeling rationale to clarify that the model space was designed to compare feature-based and integrated value-based accounts of behavior, with MDP-derived ΔQ-values serving as a normative benchmark rather than a direct process-level account of cognition.

These additions help clarify both the ecological motivation of the task and the theoretical rationale underlying the computational analyses.

Comment 2

"The modeling strategy, i.e. comparing simple heuristics, multi-heuristic policies, and optimal policies, is methodologically sound. The use of Bayesian model inference (protected exceedance probability) adds robustness. That said, the sample size (N=29) is relatively small for the complexity of the models and individual-level variability they aim to capture. A justification for such a sample is in my opinion direly needed. Power analyses or sensitivity checks would strengthen the claims."

Response:
We thank the reviewer for raising this important point. We agree that the sample size limits the precision with which inter-individual differences can be characterized and have expanded the discussion of this limitation in the revised manuscript.

At the same time, the study is based on a large number of repeated sequential decisions (10,270 decisions overall), making it particularly well suited for hierarchical computational modeling approaches that leverage within-subject information while estimating group-level effects. To better accommodate this data structure, we substantially revised the modeling framework relative to the original manuscript by replacing single-subject estimation with a hierarchical Bayesian approach and replacing BIC-based model comparison with PSIS-LOO cross-validation.

Because the present analyses rely on hierarchical Bayesian models fitted to sequential trial-wise data, conventional power analyses are not straightforwardly applicable. Instead, we expanded the set of robustness and sensitivity analyses commonly recommended for computational modeling studies. Specifically, we now report convergence diagnostics, posterior predictive checks, and additional robustness analyses evaluating alternative explanations for the observed effects. For example, the revised manuscript now states:

"All models showed satisfactory convergence diagnostics (all $\hat{R} < 1.01$, no divergent transitions, and effective sample sizes within recommended ranges). Posterior predictive checks further indicated that the fitted models reproduced the main patterns observed in the behavioral data (S2 Figure 5)." (Lines 489-491)

Together, these analyses suggest that the principal findings are robust and unlikely to reflect poor model fit, convergence issues, or overfitting. Furthermore, we also conducted parameter and model recoveries (see Response to Comment 3).

Nevertheless, we agree that larger samples will be important for more precisely characterizing inter-individual variability and assessing the generalizability of the observed effects, and we now mention this point more explicitly in the Limitations section.

Comment 3

"Additionally, while logistic regression and BIC-based comparisons are appropriate, more detail on prior selection or model identifiability safeguards (e.g., parameter recovery diagnostics and other practices such as those outlined in Palminteri et al 2017 or Wilson et al 2019) could help reassure readers of statistical rigor."

Response:
We thank the reviewer for this important suggestion. In response, we substantially expanded the methodological description of the modeling framework and added details regarding prior specification, hierarchical estimation, and model identifiability safeguards, following recommendations for computational modeling studies (e.g., Palminteri et al., 2017; Wilson & Collins, 2019).

Because the revised manuscript replaces the original single-subject maximum-likelihood framework with hierarchical Bayesian estimation, we now explicitly describe the prior distributions, partial pooling structure, non-centered parameterization, sampling settings, and predictor standardization procedures. For example, the revised manuscript now states:

"All models were fit using hierarchical Bayesian logistic regression using the python package PyMC (...). Group-level regression coefficients were assigned weakly informative normal priors centered at zero (N(0,1)). Variance parameters governing subject-level variability were assigned Exp(1) priors. Subject-level effects were estimated hierarchically using non-centered parameterization to improve sampling efficiency and stabilize No-U-Turn Sampler (NUTS) estimation. (...) Unless otherwise specified, models were estimated using 4 independent Markov chains with 1000 tuning iterations and 1000 posterior sampling iterations per chain (target_accept = 0.90). All continuous predictors were z-scored prior to model fitting to facilitate parameter estimation, comparability across features, and the use of weakly informative priors on a common scale. For the multi-feature policy model, only continuous predictors (p(gain) and r(threat)) were standardized, while binary predictors ("wait when safe" and "binary energy state") remained unscaled. The multi-feature model additionally included fixed pairwise interaction terms between predictors, while retaining random slopes for the corresponding main effects." (Lines 465-480)

To further address model identifiability, we added both model and parameter recovery analyses. The model recovery (Figure 5) analysis evaluated whether candidate models could be reliably distinguished when fit to synthetic data generated from each model, whereas the parameter recovery (S2 Figures 3-4) analysis assessed whether parameters of the winning model could be accurately recovered from simulated datasets. These analyses demonstrated high model discriminability and acceptable parameter recoverability, providing additional evidence that the reported model-comparison results are unlikely to reflect model confusability or poor parameter identifiability.

Together, these additions substantially strengthen the transparency and statistical rigor of the modeling framework and provide safeguards against common identifiability concerns in computational modeling studies.

Comment 4

"Of note, with numerous (!) models being compared, is there any correction for multiple comparisons, or is this handled adequately by the Bayesian approach?"

Response:
We thank the reviewer for raising this point. No additional correction for multiple comparisons was applied because the model comparison does not rely on a series of independent null-hypothesis significance tests. Instead, all candidate models are evaluated jointly using PSIS-LOO cross-validation and ranked according to their relative out-of-sample predictive performance. Consequently, model selection is based on a single model-comparison framework rather than multiple pairwise statistical tests requiring family-wise error correction.

Results

Comment 1

"The results are presented clearly, with appropriate figures supporting the key claims. The finding that participants generally relied on mixed heuristic strategies but shifted toward more optimal policies under higher threat (avoidance) conditions is both intuitive and well demonstrated through the regression and model comparison analyses."

Response:
We thank the reviewer for this positive evaluation of the Results section and analytical approach. While the revised analyses remain fully consistent with the core findings of the original manuscript, they led us to refine the interpretation of the observed contextual effects. Rather than interpreting avoidance contexts as producing a global shift toward a more "optimal" or sophisticated decision strategy, the revised manuscript now emphasizes context-dependent modulation of decision-relevant features together with increased alignment to the integrated value structure of the task.

"Regarding policy modulation by condition, there was no evidence for a global shift in overall decision policy, as the main effect of condition was not reliably different from zero (...). This indicates that contextual differences in behavior cannot be explained by a uniform change in response tendencies, but instead reflect selective feature reweighting, that is, context-dependent changes in how decision-relevant features influence choice. (…)" (Lines 182-190)

At the same time, condition modulated sensitivity to the integrated decision variable ΔQ:

"Critically, condition also modulated sensitivity to the integrated decision variable ΔQ (...), with choices in avoidance contexts showing greater alignment with the integrated value structure of the task (...). (...)" (Lines 191-198)

Together, these findings led us to reinterpret the observed context effects in terms of selective modulation of decision-relevant features and increased alignment with the integrated value structure of the task, rather than a shift toward a qualitatively distinct decision strategy.

Comment 2

"The evidence for two subgroups (one heuristic, one more optimal) is particularly interesting, though it could be made more explicit statistically, e.g., via clustering or latent mixture modeling rather than descriptive inference from Bayes factors. Some aspects (e.g., differences between PEP and BF metrics) are conceptually heavy and need some serious unpacking if the paper is targeted to non-specialists, otherwise it's almost a black-box effect (a schematic or summary table of participant-level model wins would help)."

Response:

We thank the reviewer for this thoughtful suggestion. In the revised manuscript, we substantially revised the modeling framework and no longer interpret the results in terms of discrete participant subgroups or qualitatively distinct strategy classes. Consequently, the primary motivation for clustering or latent-mixture approaches no longer applies.

Instead, the revised analyses focus on how integrated value signals (ΔQ) and decision-relevant features jointly contribute to behavior within a unified hierarchical framework. These analyses indicate that contextual effects are best explained by selective modulation of feature weights together with increased alignment to the integrated value structure of the task, rather than by sharply separable heuristic and "optimal" subgroups.

To further examine inter-individual variability, we additionally included an exploratory hierarchical policy-arbitration analysis (S2 Figure 6). Consistent with the revised interpretation, arbitration weights formed a continuous distribution across participants rather than discrete clusters, suggesting graded variation in policy alignment rather than distinct participant groups.

We also agree that the original presentation of participant-level Bayes factors and protected exceedance probabilities was conceptually dense. Accordingly, the revised manuscript places substantially greater emphasis on the unified hierarchical analyses, which provide a more direct and interpretable account of the observed behavioral effects.

Comment 3

"Am I correct to understand that there were 72 forests over multiple blocks? I wonder if participants showed learning, or fatigue effects, across the experiment? This could interact with the main findings and should at the very least be acknowledged."

Response:
Participants completed 72 forests across four experimental blocks, making potential learning and fatigue effects an important consideration. In response, we explicitly incorporated block number and its interactions with decision features into the revised hierarchical modeling framework to assess experience-dependent changes across the experiment.

These analyses revealed systematic behavioral changes across blocks, including an overall reduction in foraging behavior and increased sensitivity to ΔQ-values over time. The revised manuscript now states:

"Across blocks, participants showed systematic shifts in behavior, characterized by an overall reduction in foraging (...) and increased alignment with optimal value differences (...). (...)" (Lines 201-203)

Importantly, the critical condition-dependent effects remained present after accounting for these block-related changes, indicating that the observed context effects cannot be explained solely by learning or fatigue over the course of the experiment.

We additionally included block number as a covariate in the exploratory policy-arbitration analysis and now discuss the remaining interpretational limitations regarding learning, adaptation, and fatigue more explicitly in the revised manuscript.

Discussion

Comment 1

"The discussion is comprehensive and ties the findings neatly back to theoretical frameworks of bounded rationality and meta-decision making. The authors do well to highlight context-dependent shifts in cognitive control and link their findings to both reinforcement learning theory and behavioral ecology. The emphasis on inter-individual variability and links to psychopathology is a strong addition and gives the paper translational depth."

Response:
We thank the reviewer for this encouraging assessment of the Discussion section and theoretical framing. In the revised manuscript, we further refined the conceptual interpretation of the findings, particularly regarding the relationship between feature-based decision processes, integrated value representations, and adaptive behavior under approach–avoidance conflict.

A central change was to more clearly distinguish alignment with normative value signals from the computational mechanisms that generate behavior. Rather than interpreting avoidance contexts as producing a shift toward a qualitatively distinct or more "optimal" strategy, the revised Discussion emphasizes context-dependent changes in how decision-relevant information contributes to choice. We further expanded the theoretical discussion by relating the findings to optimization-based perspectives offering simpler solutions to approximate adaptive behavior. These changes are implemented as following:

"Importantly, the influence of ΔQ explained behavior beyond the individual feature predictors. However, this does not imply that participants explicitly computed ΔQ-values or switched to a qualitatively distinct decision strategy. Rather than representing competing process-level accounts, feature-based and integrated value-based models may capture different levels of description. Feature-based models characterize the information influencing choice, whereas integrated value models characterize how that information is combined within the structure of the task. Consistent with optimization-based perspectives (e.g., Salimans et al., 2017), complex or near-optimal behavior may emerge through the tuning of relatively simple parameterized policies, whose lower-dimensional structure provides a scalable and flexible means of adaptation without requiring explicit construction of exhaustive value representations. From this perspective, greater alignment with integrated value signals may arise through adaptive changes in how decision-relevant features are weighted and combined" (Lines 284-294)

We also expanded the theoretical discussion linking the findings to boundedly optimal and optimization-based perspectives on decision-making, as well as the translational implications of adaptive feature integration and reweighting for understanding individual differences and maladaptive behavior (Discussion, lines 322–331).

Comment 2

"However, the discussion could benefit from a more explicit acknowledgment of methodological limitations. In particular, the modest sample, potential fatigue effects in long sequential tasks, and the artificiality of the threat framing (no real aversive outcomes, which is a paramount limitation that could even be considered a make-or-brake limitation if left open to interpretation). Additionally, Claims about "sophisticated" or "optimal" control should be tempered, as the MDP-derived benchmark is a theoretical ideal rather than an empirically verifiable neural process."

Response:
We thank the reviewer for highlighting these important limitations. Several of these concerns overlap with points raised before regarding sample size (Methods Comment 2), learning and fatigue effects (Results Comment 3), the symbolic nature of the threat manipulation (Introduction Comment 3), and the interpretation of alignment with MDP-derived policies. We also substantially revised and expanded the Limitations section to address each of these issues more explicitly and cautiously (Discussion, lines 331-366).

Regarding sample size, we now state:

"First, the sample comprised 29 participants. Although each participant contributed a large number of repeated decisions and all models were estimated hierarchically, larger samples will be needed to establish the robustness and generalizability of the present findings." (Lines 306-308)

Regarding the symbolic nature of the threat manipulation, we now state:

"Second, the manipulation of threat was purely symbolic and not validated with physiological or subjective measures, leaving open whether the avoidance condition induced genuine affective responses or instead reflected differences in attention or task demands. Furthermore, the task remains an abstract and simplified environment, and it is unclear how well the observed patterns generalize to real-world decision-making involving more salient or consequential threats." (Lines 308-313)

We also addressed the optimal policy representing a normative benchmark, rather than a process level model in the Limitations:

"Third, the optimal policy serves as a normative benchmark rather than a process-level account; thus, increased alignment with ΔQ-values should not be interpreted as evidence that participants explicitly compute or implement this policy, but may instead reflect improved integration of decision-relevant features or reduced decision noise." (Lines 342-346)

Finally, we now discuss learning, adaptation, and fatigue effects more explicitly:

"Finally, while we accounted for block-related changes, the present framework does not fully dissociate the computational mechanisms underlying these effects. In particular, the observed ΔQ-values × block interaction indicates that behavior changed systematically across the experiment, but the current modeling approach remains descriptive and does not specify whether these changes reflect learning, strategic adaptation, fatigue, attentional shifts, or other latent processes." (Lines 333-337)

Reviewer #2

"This manuscript introduces a novel sequential "hunter-gatherer" foraging task that elegantly operationalizes approach–avoidance conflict by dissociating starvation and predation risks. Using a model-based framework ranging from simple heuristics to an optimal Markov Decision Process (MDP), the authors report that participants shift toward more sophisticated (i.e., near-optimal) decision policies under high-threat (avoidance) conditions. The findings are interpreted as evidence for context-dependent meta-decision making, wherein cognitive resources are dynamically allocated based on environmental stakes.

The experimental design is creative and ecologically grounded, offering a valuable departure from

static choice paradigms. The computational modeling pipeline is transparent, well-documented, and publicly shared—exemplifying best practices in open science. The distinction between boundary conditions (e.g., BES, WWS) and trade-off trials is thoughtful and strengthens the validity of model comparisons."

Respone:
We sincerely thank Reviewer #2 for the careful, detailed, and highly constructive evaluation of our manuscript. We particularly appreciate the reviewer's positive assessment of the ecological task design, the computational modeling framework, and the distinction between boundary-state conditions and trade-off trials. The reviewer's comments substantially improved the conceptual clarity, methodological rigor, and interpretational precision of the work.

In response to the review, we substantially revised both the analytical framework and the theoretical interpretation of the findings. Key changes include the implementation of a hierarchical Bayesian modeling framework with predictive model comparison, the addition of recovery, robustness, and control analyses, and a more explicit distinction between normative optimality and process-level inference. We also refined the conceptual framing of the results. Rather than interpreting avoidance contexts as producing a shift toward a more sophisticated or qualitatively distinct decision strategy, the revised manuscript emphasizes context-dependent modulation of decision-relevant features together with increased alignment to the integrated value structure of the task. Finally, we expanded the discussion of alternative explanations, learning effects, task difficulty, and methodological limitations. The specific changes made in response to each comment are detailed below.

Comment 1

"Threat manipulation lacks empirical validity and reliability. This is the core flaw as I see. The "predator" is represented only by a red border, with no physiological (e.g., skin conductance, heart rate) or subjective (e.g., fear/anxiety ratings) measures to confirm that it induced genuine threat perception. Without such validation, the observed behavioral shift could equally reflect increased cognitive load or task complexity rather than adaptive upregulation under threat."

Response:
We thank the reviewer for raising this important concern. We agree that the present study does not establish whether the avoidance condition elicited genuine affective threat responses comparable to those induced by validated physiological or subjective threat manipulations. Consequently, we have substantially revised the manuscript to more clearly delimit the scope of our conclusions and explicitly discuss this issue as a limitation.

Importantly, the primary objective of the present study was not to investigate the phenomenology or physiology of threat, but rather the computational mechanisms through which behavior adapts when reward-related and threat-related information compete during sequential decision-making. To make this focus more explicit, we substantially revised the Introduction. In particular, we now state:

"Integrating sequential decision-making with AAC is particularly valuable because adaptive behavior requires both anticipating future consequences and balancing competing gains and risks. Sequential choice paradigms provide a powerful framework for studying these processes because they allow behavior to be analyzed in terms of decision policies (...). Such policies may depend either on integrated value computations or on simpler decision-relevant features derived from observable environmental cues, such as reward probability or threat risk. (...) Distinguishing between behavior that arises from integrated value representations and behavior that emerges

through adaptive weighting of decision-relevant features is important because superficially similar adaptive behavior may arise from fundamentally different computational mechanisms, with distinct implications for theories of decision-making in psychology and economics." (Lines 13-39)

We also clarified how this question was operationalized within the computational modeling framework:

"By combining these components, the multi-feature policy provides a structured, low-dimensional approximation of the optimal policy that captures both environmental dynamics and internal resource constraints without requiring explicit forward planning. This formulation allows us to test whether adaptive behavior is better explained by structured feature integration, isolated decision variables, or exhaustive MDP-based evaluations. Importantly, the resulting model space allows us to determine whether apparent alignment with optimal behavior is better explained by integrated value representations or by selective modulation of individual decision features." (Lines 148-154)

At the same time, we agree that the symbolic nature of the manipulation limits strong conclusions regarding affective threat processing and ecological validity. We therefore now explicitly state in the Limitations section:

"Second, the manipulation of threat was purely symbolic and not validated with physiological or subjective measures, leaving open whether the avoidance condition induced genuine affective responses or instead reflected differences in attention or task demands. Furthermore, the task remains an abstract and simplified environment, and it is unclear how well the observed patterns generalize to real-world decision-making involving more salient or consequential threats." (Lines 308-313)

Together, these revisions more clearly distinguish computational adaptation from affective threat processing, while clarifying both the scope of our conclusions and the limitations of the present threat manipulation.

Comment 2

"Sample size is insufficient for robust individual-level inference. With N = 29, the study is underpowered to characterize heterogeneity in strategy use. The claim that "some individuals" adopt more optimal policies in avoidance contexts is intriguing but remains speculative without systematic analysis of moderating traits (e.g., anxiety, working memory). Moreover, model comparison metrics (e.g., PEP) may be unduly influenced by a few high-performing outliers, as suggested by Figure 5B."

Response:
We thank the reviewer for raising this important point. We agree that the sample size limits the strength of conclusions regarding inter-individual variability and have revised both the modeling framework and the corresponding interpretation accordingly.

At the same time, the study is based on a large number of repeated sequential decisions (10,270 decisions overall), making it particularly well suited for hierarchical computational modeling approaches that leverage within-subject information while estimating group-level effects. To better accommodate this data structure, we substantially revised the modeling framework relative to the original manuscript by replacing single-subject estimation with a hierarchical Bayesian approach and replacing BIC-based model comparison with PSIS-LOO cross-validation.

Most importantly, the revised manuscript no longer interprets the results in terms of distinct behavioral subgroups or qualitatively different strategy classes. Instead, all models are now estimated within a hierarchical Bayesian framework that leverages the large number of repeated decisions contributed by each participant while stabilizing inference at the group level. As we now state:

"Because participants contributed a large number of repeated decisions, all computational models were estimated using a hierarchical Bayesian framework. This approach pools information across participants while preserving individual differences, allowing group-level and subject-level effects to be estimated simultaneously. By leveraging the hierarchical structure of the data, hierarchical models improve statistical efficiency and stabilize parameter estimates, particularly in repeated-measures designs with many observations per participant." (Lines 155-160)

Furthermore, the revised analyses no longer support the original interpretation in terms of heuristic versus "optimal" participant subgroups. Instead, the hierarchical analyses indicate a group-level increase in alignment with the integrated value structure of the task under avoidance conditions.

To examine the possibility of inter-individual variability, we additionally conducted an exploratory hierarchical policy-arbitration analysis (S2 Figure 6). Consistent with the revised interpretation, arbitration weights formed a continuous distribution across participants rather than discrete clusters, suggesting graded variation in policy alignment rather than distinct participant groups. Moreover, the analysis largely reproduced the main findings, indicating that the observed effects were not driven by a small number of high-performing individuals or by discrete shifts between competing policy classes.

We agree that larger samples will be needed to more precisely characterize individual differences and identify potential moderators of context-dependent adaptation. Accordingly, we now state in the Limitations section:

"First, the sample comprised 29 participants. Although each participant contributed a large number of repeated decisions and all models were estimated hierarchically, larger samples will be needed to establish the robustness and generalizability of the present findings." (Lines 333-335)

Together, these revisions substantially temper claims regarding individual differences while providing a more appropriate basis for group-level inference from the present dataset.

Comment 3

"Limited computational or theoretical novelty. While the task is innovative, the core finding – that threat promotes more deliberative/model-based control – largely replicates established principles from dual-process theory and behavioral ecology. The paper does not propose a new algorithmic mechanism, formalize how policy selection is implemented, or offer a generative model of meta-decision making beyond post hoc model fitting."

Response:
We thank the reviewer for this thoughtful comment. We agree that the present work does not propose a novel algorithmic mechanism for meta-decision making, nor does it provide a generative model specifying how policy selection is implemented. However, one of the major revisions of the manuscript was a shift away from the original interpretation in terms of increased "policy sophistication" under threat and toward a more specific mechanistic question: whether contextual

adaptation is better explained by changes in integrated value representations or by context-dependent modulation of decision-relevant features.

To make this distinction explicit, we substantially revised the Introduction. In particular, we now state:

"Importantly, behavior need not rely on explicit value maximization to appear near-optimal. Similar adaptive behavior may also emerge through selective weighting of informative decision features, that is, through changes in how strongly decision-relevant features influence choice, allowing adaptive policies to be approximated without explicit computation of exhaustive value representations. Such lower-dimensional policy representations may provide a computationally scalable and flexible basis for adaptation (...)." (Lines 30-35)

Accordingly, the revised manuscript focuses on the computational mechanisms through which adaptive behavior emerges:

"Within AAC, contextual variation emerges from how reward and threat jointly determine the long-term value of available actions. (...) Recent accounts suggest that this flexibility may arise not through switches between entirely distinct decision strategies, but through context-dependent changes in reliance on specific decision features (...). Under this view, approach and avoidance contexts may shape behavior by selectively modulating the relative influence of specific decision features, such as reward probability or threat risk, while preserving a shared underlying computational process." (Lines 40-51)

"(...) (1) whether approach and avoidance contexts differ in the extent to which behavior aligns with optimal policies, and (2) whether such differences can be explained by context-dependent modulation of decision-relevant features." (Lines 66–69)

To address this question, we substantially revised the analytical framework. The revised hierarchical analyses directly examined how individual decision features and integrated value signals jointly contribute to behavior. The resulting analyses showed that choices were influenced by multiple decision-relevant features spanning reward, threat, and state-dependent boundary conditions. Importantly, avoidance contexts selectively modulated specific feature weights, most notably reducing the influence of reward probability, while simultaneously increasing alignment with the integrated value structure of the task (stat-action value: $\Delta Q = Q_{foraging} - Q_{waiting}$) (Results, lines 190–249).

These findings led to a substantially revised interpretation of the results. As we now state in the Discussion:

"Importantly, the influence of ΔQ explained behavior beyond the individual feature predictors. However, this does not imply that participants explicitly computed ΔQ-values or switched to a qualitatively distinct decision strategy. Rather than representing competing process-level accounts, feature-based and integrated value-based models may capture different levels of description. Feature-based models characterize the information influencing choice, whereas integrated value models characterize how that information is combined within the structure of the task. Consistent with optimization-based perspectives (...), complex or near-optimal behavior may emerge through the tuning of relatively simple parameterized policies (...). From this perspective, greater alignment with integrated value signals may arise through adaptive changes in how decision-relevant features are weighted and combined." (Lines 284-294)

We therefore view the primary contribution of the manuscript not as demonstrating that threat promotes deliberative or model-based control. Rather, the contribution lies in identifying context-dependent feature modulation and integration as a candidate computational mechanism through which adaptive behavior emerges in sequential approach–avoidance conflicts. At the same time, we agree that developing explicit generative models capable of explaining how such feature reweighting emerges remains an important direction for future research.

Comment 4

"Conceptual conflation between normative optimality and psychological process. Equating alignment with the MDP-derived optimal policy with "more sophisticated" or "deliberative" cognition is problematic. Humans may approximate optimal behavior via entirely different mechanisms (e.g., sampling, analogy, or evolved heuristics). The authors do not rule out simpler explanations, such as reduced response noise or increased attention under threat (i.e., bounded rationality with higher inverse temperature)."

Response:
We thank the reviewer for raising this important conceptual concern. We agree that alignment with an MDP-derived optimal policy should not be interpreted as direct evidence for explicit model-based computation, deliberative reasoning, or implementation of the optimal policy itself. Indeed, one of the central goals of the revision was to more clearly distinguish normative benchmarks from process-level explanations of behavior.

To make this distinction explicit, we revised the Introduction to clarify the role of the optimal policy within the present study:

"Policies based on integrated values (...) can be formalized within Markov Decision Processes (MDPs), which specify value-maximizing behavior across temporally extended choice sequences (...). Within such frameworks, optimal policies can be derived using dynamic programming (...), yielding state–action values (Q-values) that quantify the expected long-term value of alternative actions and provide a normative benchmark against which observed behavior can be evaluated." (Lines 21-26)

We further clarified this point when introducing the task:

"The task was designed according to a fully specified MDP, allowing us to mathematically derive the optimal policy as a normative benchmark." (Lines 73-74)

Accordingly, the revised analyses focus on the relationship between integrated value signals and decision-relevant features as alternative computational descriptions of adaptive behavior (see Response to Comment 3).

Finally, we now explicitly acknowledge this limitation in the Discussion:

"Third, the optimal policy serves as a normative benchmark rather than a process-level account; thus, increased alignment with ΔQ-values should not be interpreted as evidence that participants explicitly compute or implement this policy, but may instead reflect improved integration of decision-relevant features or reduced decision noise." (Lines 315-319)

We also agree that explanations such as increased attention, reduced response noise, or other low-dimensional changes in behavior remain plausible. Accordingly, the revised manuscript focuses on

the observed behavioral pattern itself: increased alignment with the integrated value structure of the task together with context-dependent modulation of decision-relevant features.

Comment 5

"Ambiguity in condition matching. Although the authors report comparable survival rates across conditions, the joint success probability (p(success)) is slightly higher in avoidance forests (0.37 vs. 0.30). Even if statistically non-significant, this imbalance could confound interpretations: participants might appear "more optimal" simply because the environment was marginally more favorable, not because they employed more complex strategies."

Response:
We thank the reviewer for raising this important point. We agree that differences in environmental structure represent a potential concern when interpreting condition effects in sequential decision-making tasks. In particular, we acknowledge that avoidance forests showed a somewhat higher average joint success probability than approach forests. Although survival rates did not differ significantly between conditions, we agree that survival rates alone do not fully rule out differences in task difficulty.

For this reason, the revised manuscript includes several additional analyses specifically aimed at evaluating whether the observed condition effects could be explained by differences in overall task difficulty rather than by changes in how information was used to guide behavior.

First, we directly characterized the theoretical decision space of the task using MDP-derived state–action value differences (ΔQ), which served as a measure of choice difficulty. The revised Results section now states:

"This pattern was highly similar across approach and avoidance conditions (Figure 3B–C), suggesting comparable theoretical difficulty across contexts. Consistent with this, a Kolmogorov–Smirnov test revealed only a small difference between the ΔQ distributions (D = 0.04), despite statistical significance driven by the large number of theoretically possible states (p < .01)." (Lines 119-123)

Second, we examined response times as an independent indicator of processing demands. As reported in the revised Results:

"(...) There was no difference in RTs between conditions (...). We next examined whether response times varied as a function of decision difficulty by regressing RTs on absolute state–action value differences (|ΔQ|), condition, and their interaction. No effects of |ΔQ| (...), condition (...), or their interaction (...) were observed. (...)" (Lines 127-134)

Third, the increased influence of ΔQ under avoidance persisted after residualization analyses removing variance shared with the multi-feature predictors (S2 Figure 1B). In addition, an exploratory policy-arbitration analysis (S2 Figure 6) produced a similar pattern of results while explicitly accounting for overlap between competing policy representations, suggesting that the observed increase in ΔQ-alignment cannot be readily explained by policy overlap alone.

At the same time, we agree that this concern cannot be ruled out completely. Accordingly, we now explicitly discuss this issue in the Limitations section:

"Importantly, although alternative explanations for the ΔQ-values × condition interaction, such as differences in task difficulty or cognitive demand, cannot be fully excluded, several analyses argue

against a simple ‘easier avoidance condition’ account. (...) Moreover, response times did not differ as a function of decision difficulty or context, and the increased influence of ΔQ under avoidance persisted even when ΔQ-values were residualized with respect to the multi-feature model predictors. (...)” (Lines 320-332)

Taken together, we agree that the environmental conditions were not perfectly matched on every descriptive statistic. However, the convergence of survival-rate analyses, ΔQ-based difficulty analyses, response-time analyses, residualization controls, policy-arbitration analyses, and the explicit limitations discussion suggests that the observed increase in ΔQ-alignment under avoidance is unlikely to be explained solely by a marginally more favorable environment.
