# Supplementary material for: Context-dependent feature modulation shapes human decision policies in approach–avoidance conflicts
Source: PLoS Comput Biol. 2026 Sep 30;22(9):e1014793. doi: 10.1371/journal.pcbi.1014793 (PMC13652344; doi:10.1371/journal.pcbi.1014793)
Supplement: Peer review history

# Peer review: Decision Letter 0

**DOI:** 10.1371/journal.pcbi.1014793.r001

**Authors:** Feilim Mac Gabhann, Zhanshan (Sam) Ma

17 Mar 2026

Humans employ more sophisticated decision policies when threat is higher in approach-avoidance conflicts.

PLOS Computational Biology

Dear Dr. Korn,

Thank you for submitting your manuscript to PLOS Computational Biology. After careful consideration, we feel that it has merit but does not fully meet PLOS Computational Biology's publication criteria as it currently stands. Therefore, we invite you to submit a revised version of the manuscript that addresses the points raised during the review process.

Please submit your revised manuscript by May 17 2026 11:59PM. If you will need more time than this to complete your revisions, please reply to this message or contact the journal office at ploscompbiol@plos.org. When you're ready to submit your revision, log on to https://www.editorialmanager.com/pcompbiol/ and select the 'Submissions Needing Revision' folder to locate your manuscript file.

Please include the following items when submitting your revised manuscript:

* A letter that responds to each point raised by the editor and reviewer(s). You should upload this letter as a separate file labeled 'Response to Reviewers'. This file does not need to include responses to formatting updates and technical items listed in the 'Journal Requirements' section below.

* A marked-up copy of your manuscript that highlights changes made to the original version. You should upload this as a separate file labeled 'Revised Manuscript with Track Changes'.

* An unmarked version of your revised paper without tracked changes. You should upload this as a separate file labeled 'Manuscript'.

If you would like to make changes to your financial disclosure, competing interests statement, or data availability statement, please make these updates within the submission form at the time of resubmission. Guidelines for resubmitting your figure files are available below the reviewer comments at the end of this letter

We look forward to receiving your revised manuscript.

Kind regards,

Zhanshan (Sam) Ma, Ph.D

Academic Editor

PLOS Computational Biology

Feilim Mac Gabhann

Editor-in-Chief

PLOS Computational Biology

**Additional Editor Comments (if provided):**

**Journal Requirements:**

If the reviewer comments include a recommendation to cite specific previously published works, please review and evaluate these publications to determine whether they are relevant and should be cited. There is no requirement to cite these works unless the editor has indicated otherwise.

[Note: HTML markup is below. Please do not edit.]

**Reviewers' comments:**

Reviewer's Responses to Questions

**Comments to the Authors:**

**Please note here if the review is uploaded as an attachment.**

Reviewer #1: I would like to thank the editor for the opportunity to review this manuscript. The study tackles a timely and important question about how humans dynamically adjust their decision-making strategies in threatening versus neutral environments. By combining an ecologically motivated approach–avoidance paradigm with formal model comparison, this work addresses a significant gap in understanding how environmental threat modulates policy sophistication. While I find the approach valid and original, I have some minor reservations concerning actual sample size and general robustness checks. More detailed comments follow below:

INTRODUCTION

(1)The introduction is conceptually strong and well written. It clearly situates the study within the context of model-based versus model-free decision-making and frames the question through an ecological and evolutionary lens.

(2)The authors do an excellent job connecting dual-process frameworks with behavioral ecology concepts such as bounded rationality and meta-decision making.

(3) Still, I must point out that I personally find this introduction generally dense, and somewhat dissagregated regarding the other topics it touches upon. The flow between the reinforcement learning literature (Daw et al.) and ecological foraging literature (Kolling, Mobbs, etc.) could be more cohesive, since at times these threads feel like parallel lines rather than something that has been theoretically integrated.

(4) While the hypothesis is clearly stated (if I follow correctly, that one should anticipate “greater policy sophistication" under threat), the whole section might benefit from an explicit operational definition of “sophistication” earlier on, i.e. whether this refers to model-based computation, policy flexibility, or response adaptation.

Overall, the introduction effectively motivates the study, but pruning repetition and clarifying the conceptual bridge between MDP modeling and human behavioral ecology would improve accessibility.

METHODS

(1) The methodological design is thoughtful and rigorous. The “hunter-gatherer” paradigm is a creative and ecologically valid extension of previous work by Korn & Bach. The MDP formalization is well justified, and the implementation of “approach” and “avoidance” forests is elegant in manipulating threat without confounding motivation and reward structure.

(2) The modeling strategy, i.e. comparing simple heuristics, multi-heuristic policies, and optimal policies, is methodologically sound. The use of Bayesian model inference (protected exceedance probability) adds robustness. That said, the sample size (N=29) is relatively small for the complexity of the models and individual-level variability they aim to capture. A justification for such a sample is in my opinion direly needed. Power analyses or sensitivity checks would strengthen the claims.

(3) Additionally, while logistic regression and BIC-based comparisons are appropriate, more detail on prior selection or model identifiability safeguards (e.g., parameter recovery diagnostics and other practices such as those outlined in Palminteri et al 2017 or Wilson et al 2019) could help reassure readers of statistical rigor.

(4) Of note, with numerous (!) models being compared, is there any correction for multiple comparisons, or is this handled adequately by the Bayesian approach?

(References for this section:

-The Importance of Falsification in Computational Cognitive Modeling, Stefano Palminteri, Valentin Wyart, Etienne Koechlin 2017

-Ten simple rules for the computational modeling of behavioral data Robert C Wilson, Anne Ge Collins, 2019)

RESULTS

(1)The results are presented clearly, with appropriate figures supporting the key claims. The finding that participants generally relied on mixed heuristic strategies but shifted toward more optimal policies under higher threat (avoidance) conditions is both intuitive and well demonstrated through the regression and model comparison analyses.

(2)The evidence for two subgroups (one heuristic, one more optimal) is particularly interesting, though it could be made more explicit statistically, e.g., via clustering or latent mixture modeling rather than descriptive inference from Bayes factors. Some aspects (e.g., differences between PEP and BF metrics) are conceptually heavy and need some serious unpacking if the paper is targeted to non-specialists, otherwise it's almost a black-box effect (a schematic or summary table of participant-level model wins would help).

(3) Am I correct to understand that there were 72 forests over multiple blocks ? I wonder if participants showed learning, or fatigue effects, across the experiment? This could interact with the main findings and should at the very least be acknowledged.

DISCUSSION

(1) The discussion is comprehensive and ties the findings neatly back to theoretical frameworks of bounded rationality and meta-decision making. The authors do well to highlight context-dependent shifts in cognitive control and link their findings to both reinforcement learning theory and behavioral ecology. The emphasis on inter-individual variability and links to psychopathology is a strong addition and gives the paper translational depth.

(2) However, the discussion could benefit from a more explicit acknowledgment of methodological limitations. In particular, the modest sample, potential fatigue effects in long sequential tasks, and the artificiality of the threat framing (no real aversive outcomes, which is a paramount limitation that could even be considered a make-or-brake limitation if left open to interpretation). Additionally, Claims about “sophisticated” or “optimal” control should be tempered, as the MDP-derived benchmark is a theoretical ideal rather than an empirically verifiable neural process.

Reviewer #2: This manuscript introduces a novel sequential “hunter-gatherer” foraging task that elegantly operationalizes approach–avoidance conflict by dissociating starvation and predation risks. Using a model-based framework ranging from simple heuristics to an optimal Markov Decision Process (MDP), the authors report that participants shift toward more sophisticated (i.e., near-optimal) decision policies under high-threat (avoidance) conditions. The findings are interpreted as evidence for context-dependent meta-decision making, wherein cognitive resources are dynamically allocated based on environmental stakes.

The experimental design is creative and ecologically grounded, offering a valuable departure from static choice paradigms. The computational modeling pipeline is transparent, well-documented, and publicly shared—exemplifying best practices in open science. The distinction between boundary conditions (e.g., BES, WWS) and trade-off trials is thoughtful and strengthens the validity of model comparisons. Despite these strengths, I have several serious concerns that limit the manuscript’s suitability for publication in its current form:

Threat manipulation lacks empirical validity and reliability. This is the core flaw as I see. The “predator” is represented only by a red border, with no physiological (e.g., skin conductance, heart rate) or subjective (e.g., fear/anxiety ratings) measures to confirm that it induced genuine threat perception. Without such validation, the observed behavioral shift could equally reflect increased cognitive load or task complexity rather than adaptive upregulation under threat.

Sample size is insufficient for robust individual-level inference. With N = 29, the study is underpowered to characterize heterogeneity in strategy use. The claim that “some individuals” adopt more optimal policies in avoidance contexts is intriguing but remains speculative without systematic analysis of moderating traits (e.g., anxiety, working memory). Moreover, model comparison metrics (e.g., PEP) may be unduly influenced by a few high-performing outliers, as suggested by Figure 5B.

Limited computational or theoretical novelty. While the task is innovative, the core finding—that threat promotes more deliberative/model-based control—largely replicates established principles from dual-process theory and behavioral ecology. The paper does not propose a new algorithmic mechanism, formalize how policy selection is implemented, or offer a generative model of meta-decision making beyond post hoc model fitting.

Conceptual conflation between normative optimality and psychological process. Equating alignment with the MDP-derived optimal policy with “more sophisticated” or “deliberative” cognition is problematic. Humans may approximate optimal behavior via entirely different mechanisms (e.g., sampling, analogy, or evolved heuristics). The authors do not rule out simpler explanations, such as reduced response noise or increased attention under threat (i.e., bounded rationality with higher inverse temperature).

Ambiguity in condition matching. Although the authors report comparable survival rates across conditions, the joint success probability (p(success)) is slightly higher in avoidance forests (0.37 vs. 0.30). Even if statistically non-significant, this imbalance could confound interpretations: participants might appear “more optimal” simply because the environment was marginally more favorable, not because they employed more complex strategies.

**********

**Have the authors made all data and (if applicable) computational code underlying the findings in their manuscript fully available?**

The PLOS Data policy requires authors to make all data and code underlying the findings described in their manuscript fully available without restriction, with rare exception (please refer to the Data Availability Statement in the manuscript PDF file). The data and code should be provided as part of the manuscript or its supporting information, or deposited to a public repository. For example, in addition to summary statistics, the data points behind means, medians and variance measures should be available. If there are restrictions on publicly sharing data or code —e.g. participant privacy or use of data from a third party—those must be specified.

Reviewer #1: Yes

Reviewer #2: None

**********

PLOS authors have the option to publish the peer review history of their article (what does this mean?). If published, this will include your full peer review and any attached files.

If you choose “no”, your identity will remain anonymous but your review may still be made public.

**Do you want your identity to be public for this peer review?**  For information about this choice, including consent withdrawal, please see our Privacy Policy.

Reviewer #1: **Yes:**  Hernán Anlló

Reviewer #2: No

[NOTE: If reviewer comments were submitted as an attachment file, they will be attached to this email and accessible via the submission site. Please log into your account, locate the manuscript record, and check for the action link "View Attachments". If this link does not appear, there are no attachment files.]

**Figure resubmission:**

**Reproducibility:**

?>

# Author comment: Author response to Decision Letter 1

**DOI:** 10.1371/journal.pcbi.1014793.r002

24 Jun 2026

Attachment Submitted filename: AAA_R1_response.pdf

# Peer review: Decision Letter 1

**DOI:** 10.1371/journal.pcbi.1014793.r003

**Authors:** Feilim Mac Gabhann, Zhanshan (Sam) Ma

31 Aug 2026

Dear Dr. Korn,

We are pleased to inform you that your manuscript 'Context-dependent feature modulation shapes human decision policies in approach–avoidance conflicts' has been provisionally accepted for publication in PLOS Computational Biology.

Before your manuscript can be formally accepted you will need to complete some formatting changes, which you will receive in a follow up email. A member of our team will be in touch with a set of requests.

Please note that your manuscript will not be scheduled for publication until you have made the required changes, so a swift response is appreciated.

IMPORTANT: The editorial review process is now complete. PLOS will only permit corrections to spelling, formatting or significant scientific errors from this point onwards. Requests for major changes, or any which affect the scientific understanding of your work, will cause delays to the publication date of your manuscript.

Should you, your institution's press office or the journal office choose to press release your paper, you will automatically be opted out of early publication. We ask that you notify us now if you or your institution is planning to press release the article. All press must be co-ordinated with PLOS.

Thank you again for supporting Open Access publishing; we are looking forward to publishing your work in PLOS Computational Biology.

Best regards,

Zhanshan (Sam) Ma, Ph.D

Academic Editor

PLOS Computational Biology

Feilim Mac Gabhann

Editor-in-Chief

PLOS Computational Biology

***********************************************************

Reviewer's Responses to Questions

**Comments to the Authors:**

**Please note here if the review is uploaded as an attachment.**

Reviewer #1: I am content with how the authors addressed my notes.

Reviewer #2: Authors have addressed my all the concerns.

**********

**Have the authors made all data and (if applicable) computational code underlying the findings in their manuscript fully available?**

The PLOS Data policy requires authors to make all data and code underlying the findings described in their manuscript fully available without restriction, with rare exception (please refer to the Data Availability Statement in the manuscript PDF file). The data and code should be provided as part of the manuscript or its supporting information, or deposited to a public repository. For example, in addition to summary statistics, the data points behind means, medians and variance measures should be available. If there are restrictions on publicly sharing data or code —e.g. participant privacy or use of data from a third party—those must be specified.

Reviewer #1: Yes

Reviewer #2: None

**********

PLOS authors have the option to publish the peer review history of their article (what does this mean?). If published, this will include your full peer review and any attached files.

If you choose “no”, your identity will remain anonymous but your review may still be made public.

**Do you want your identity to be public for this peer review?**  For information about this choice, including consent withdrawal, please see our Privacy Policy.

Reviewer #1: **Yes:**  Hernan Anllo

Reviewer #2: No

# Editor report: Acceptance letter

**DOI:** 10.1371/journal.pcbi.1014793.r004

**Authors:** Feilim Mac Gabhann, Zhanshan (Sam) Ma

PCOMPBIOL-D-25-01785R1

Context-dependent feature modulation shapes human decision policies in approach–avoidance conflicts

Dear Dr Korn,

I am pleased to inform you that your manuscript has been formally accepted for publication in PLOS Computational Biology. Your manuscript is now with our production department and you will be notified of the publication date in due course.

The corresponding author will soon be receiving a typeset proof for review, to ensure errors have not been introduced during production. Please review the PDF proof of your manuscript carefully, as this is the last chance to correct any errors. Please note that major changes, or those which affect the scientific understanding of the work, will likely cause delays to the publication date of your manuscript.

Soon after your final files are uploaded, unless you have opted out, the early version of your manuscript will be published online. The date of the early version will be your article's publication date. The final article will be published to the same URL, and all versions of the paper will be accessible to readers.

For Research, Software, and Methods articles, you will receive an invoice from PLOS for your publication fee after your manuscript has reached the completed accept phase. If you receive an email requesting payment before acceptance or for any other service, this may be a phishing scheme. Learn how to identify phishing emails and protect your accounts at https://explore.plos.org/phishing.

Thank you again for supporting PLOS Computational Biology and open-access publishing. We are looking forward to publishing your work!

With kind regards,

Sharmila Kamatchi

PLOS Computational Biology | Carlyle House, Carlyle Road, Cambridge CB4 3DN | United Kingdom ploscompbiol@plos.org | Phone +44 (0) 1223-442824 | ploscompbiol.org | @PLOSCompBiol
